# An Invasion Risk Assessment of Alien Woody Species in Potential National Park Sites in Xinjiang, China, Under Climate Change

**DOI:** 10.1002/ece3.70394

**Published:** 2024-10-08

**Authors:** Fei‐Xue Zhang, Hong‐Li Li, Ji‐Zhong Wan

**Affiliations:** ^1^ The Key Laboratory of Ecological Protection in the Yellow River Basin of National Forestry and Grassland Administration, School of Ecology and Nature Conservation Beijing Forestry University Beijing China; ^2^ Key Laboratory of Mountain Surface Processes and Ecological Regulation, Institute of Mountain Hazards and Environment Chinese Academy of Sciences Chengdu China

**Keywords:** climate change, invasive woody plants, national parks, species distribution model, Xinjiang province

## Abstract

The invasion of alien woody species may have broad ecological, economic, and health impacts on ecosystems and biodiversity under climate change. Previous studies showed that disrupting the biodiversity conservation mechanisms in protected areas can seriously threaten natural ecosystems and the protection of rare and endangered species in such protected areas. However, there is currently no standard for evaluating the invasion risk of woody plants under climate change when establishing national parks in China. Therefore, we used a species distribution model to evaluate the invasion risk of 250 invasive alien woody species in potential national park sites in Xinjiang under climate change. The results indicated that the probability of forest invasion in the potential Altai Kanas National Park was determined to be significantly higher than that of the average level in Xinjiang nature reserves, both under current and future climate conditions. At the same time, the probability of invasive woody species invading coniferous forests, broad‐leaved forests, and grassland ecosystems is higher in the Altai Kanas and Tianshan potential national parks. We found that *Acer negundo*, *Robinia pseudoacacia*, and *Amorpha fruticose* in potential parks in Xinjiang have higher invasion potential and thus require heightened vigilance to stop their spread. This study contributes to the monitoring and management of national parks and provides an actionable foundation for protecting ecosystem functions and minimizing the potential risk of invasive alien species under climate change.

## Graphical abstract

We used a species distribution model to evaluate the invasion risk of 250 highly invasive alien woody species in potential national park sites in Xinjiang under climate change. The probability of invasive woody species distribution in potential park sites in Xinjiang is significantly lower than that across China. This study can contribute to the monitoring and management of national parks and can provide an actionable foundation for protecting the ecosystem functions and minimizing the risk of alien species invasion in potential national park sites in the face of climate change.

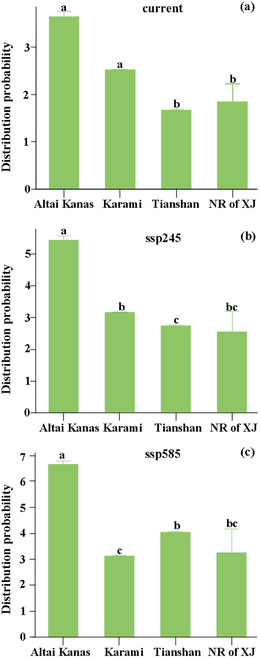

## 1 Introduction

Alien woody species may seriously harm the ecology, economy, and human well‐being of the areas they invade on a large spatial scale (Lenda et al. 2012; Monahan and Fisichelli 2017). Invasions of alien woody species have always been among the crucial issues threatening protected areas in China (Cheng et al. 2006). Alien woody species can adversely impact ecosystems and alter the ecosystem functions of invaded areas (Vila et al. 2011; Qi et al. 2014; Gallardo et al. 2016). The effective management of nature reserves to limit the invasion of woody plants can effectively protect their biodiversity, precious and endangered species, ecological balance, and other natural reserve components and resources (Bezeng et al. 2020; Liu et al. 2020). One of the most effective ways to protect biodiversity is to maximize the preservation of native species in an ecosystem, as the presence of alien woody species greatly damages the living environment of native species by occupying a larger resource space and outcompeting native species (Nunez and Medley 2011; Monahan et al. 2013). Compared to herbaceous weeds, the proportion of woody species in the lowland florae that invade nature reserves is very high (Monahan et al. 2013; Liu et al. 2020).

The climate is rapidly changing, and many national parks (81% of natural resource parks) worldwide have experienced extremely warm climate conditions compared to their historical temperature ranges (Monahan and Fisichelli 2017). Climate change is linked to measurable responses of birds, mammals, vegetation, and invasive plants within protected areas (Moritz et al. 2008; Tingley et al. 2009; Dolanc, Thorne, and Safford 2013). Climate change affects all life stages of alien woody species, including seed development, germination, and emergence, seedling growth and establishment, and ultimately, stand survival (Walck et al. 2011; Fisichelli et al. 2014).

With the acceleration of climate change and the intensification of its impact over time, broader changes are more likely to happen in terms of forest layer composition and related changes in other biological communities (Allen et al. 2010). Climate change and alien woody species invasions are two important phenomena that significantly impact global biodiversity (Dukes et al. 2009; Urban 2015). These climate shifts may affect the distribution of invasive species by changing their actual distribution areas, changing how they interact with other species, and thus impacting existing conservation and management strategies (Hellmann et al. 2008; Goncalves‐Oliveira, Rodrigues, and Benko‐Iseppon 2022). At present, many invasive alien plants have been found in various nature reserves throughout China (Liu et al. 2020a). Alien species invasion has disrupted the mechanisms by which biodiversity is protected in nature reserves, posing a serious threat to their natural ecosystems and the actual protection of rare and endangered species (Moritz et al. 2008; Wang et al. 2019). It is widely recognized that preventing an invasion is far more cost‐effective than eradicating or controlling one after it occurs (Moritz et al. 2008; Tingley et al. 2009). Therefore, predicting the impact of climate change on the distribution of alien woody species is crucial for developing action plans to restrict extinction risks (Fisichelli et al. 2014).

The seed dispersal process of invasive plants is an important component of biological invasion and is influenced by various factors, such as the bodies of dispersal, carriers, and types. The dispersal distance varies at different points and over time, and rare long‐distance dispersal events, which cannot be ignored, also occur. Previous studies have shown that the diffusion of plant seeds and propagules often requires vectors and is mostly localized, with only about 1% of species diffusion being long‐distance diffusion (LDD; Speziale et al. 2018). When there is severe plant invasion in nearby areas, the diffusion mechanisms of invasive plants, such as wind dispersal, water transmission, animal transmission, or human activities, may lead to their spread to neighboring areas (Speziale et al. 2018; Ramirez‐Albores et al. 2020; Wu et al. 2023). Seed dispersal is an important pathway for foreign plants to spread in new habitats. Of course, animals play a critical role in seed transmission, with 90% and 60% of plants in tropical and temperate regions relying on animal‐borne seed dispersal, respectively. This broad dispersal promotes plant adaptation to continuously changing environments and can enable plant invasion, promoting the introduction and colonization of invasive plants into uninvaded areas (Wu et al. 2023).

A recent global analysis has shown that invasive alien species are associated with up to 58% of extinctions of amphibian, bird, mammal, plants, and reptile species (Bellard, Cassey, and Blackburn 2016). Therefore, if invasive alien species are not properly managed, many national parks' future sustainability and value will be severely damaged (Rodrigues et al. 2004; Foxcroft et al. 2011; Thomas et al. 2012; Pyšek et al. 2013; Wan and Wang 2018). The synergistic effect between human factors, invasive alien species, and climate change complicates this task even further. A recent study found that approximately one‐third of protected areas are under severe pressure from human activities and invasive species, but areas with higher levels of protection are under less pressure from invasive plants (Jones et al. 2018). The population dynamics of incoming species inside and outside of protected nature reserves and the response of various ecosystems or communities within reserves to invasion by foreign species are important issues that must be considered when formulating nature conservation plans and strategies (Gallardo et al. 2017). Therefore, comprehensively assessing the invasion potential of invasive species and the impact of invasive species on nature reserves is of great importance for exploring conservation biology approaches and the management practices of nature reserves (Foxcroft et al. 2017).

National parks are the areas with the richest biodiversity among natural ecosystems (Colwell 2012; Li, Liu, and Lv 2019; Guo et al. 2023). National parks protect the ecological environment and its natural resources, help to develop local tourism industries, and achieve large‐scale effective protection through small‐scale and moderate development (Colwell 2012; Li, Liu, and Lv 2019). The establishment of national parks in China was initiated relatively late, and there is a substantial gap between the number of national parks in the country's eastern and western regions, as they are mainly concentrated in the southeast of China (Liu et al. 2020b). However, in the arid areas of northwestern China, national parks are urgently needed to ensure the maintenance of biodiversity and safeguard the stability of the ecosystem in the area. Xinjiang is located on the northwest border of China and has vast territories containing diverse and rich ecosystems such as snowcapped mountains, glaciers, forests, grasslands, wetlands, deserts, and the Gobi Desert (Zhang, Kung, and Johnson 2017). Establishing a national park in Xinjiang is of great importance in establishing ecological security nationwide, building an ecological security barrier along the Silk Road, and maintaining regional and national ecological security and long‐term sustainable development.

The International Union for Conservation of Nature (IUCN) stipulates that the degree of invasion of woody plants is one of the criteria for determining whether a region should be considered a national park (Dudley, Stolton, and Shadie 2008). However, there is currently no research evaluating the invasion risk of woody plants in the region, and more emphasis is placed on using the unique flora and fauna resources, geological and geomorphic resources, as well as cultural and historical characteristics of the region as the criteria for establishing national parks. However, effectively addressing the risks of sustained climate change and invasive alien species poses challenges to the establishment of national parks in Xinjiang.

In this study, we used a species distribution model to evaluate the invasion risk of woody plant species in candidate sites for Xinjiang national parks (Liang et al. 2014; Thuiller et al. 2005). The candidate park sites investigated in this study include the potential sites of Kanas National Park, Tianshan National Park, and Karamaili National Park. Establishing national parks in these sites in Xinjiang is necessary, urgent, and feasible. Karamaili, located in the east of the Junggar Basin, Xinjiang is a representative temperate arid small arbor desert ecosystem in China and the only temperate wild desert ungulate concentration area in China (Shi, Sun, and Huang 2020). The Tianshan mountain range is one of the seven major mountain range systems in the world, is the largest isolated east–west mountain range globally, and contains the largest arid region globally. It has the most typical and complete altitudinal vegetation spectrum worldwide among temperate and arid regions (Xu et al. 2012). It is also an important habitat for valuable germplasm resources of rare species such as *Panthera uncia*, northern *Capra sibirica*, and *Tetrao urogallus*. The Tianshan Mountains are also the region with among the most concentrated biodiversity in China and one of the key hotspots of global biodiversity (Xu et al. 2012; Shi et al. 2019). Kanas is located in the central region of the Altay Mountains in northern Xinjiang (Bagedeng et al. 2022; Huang et al. 2018). A national geological park is already located in the region, and it includes a glacier landform represented by the Kanas Glacier Weir. The geological heritage resources are typical, the system is well preserved, and the area boasts diverse vegetation types, which also carry high geological importance (Huang et al. 2018). The ecological value of the three potential national parks mentioned above is extremely high, and these primitive natural resources, landscape resources, and cultural conditions already meet the requirements and criteria for establishment as national parks.

In the context of climate change, the present work focused on the woody plant species with the highest invasion potential, specifically in the three candidate national park sites in Xinjiang, compared to other protected areas in China. We evaluated their invasion and distribution risks to address, prevent, and manage potential invasions. Specifically, we aimed to (1) assess the invasion risk of alien woody species in three potential national park sites in Xinjiang under climate change; (2) explore which ecosystems in potential national parks are more susceptible to invasive alien woody species; (3) identify the major alien woody species that could pose the highest invasion threat in the potential park sites in Xinjiang under the current and future climate conditions. Our results provide a necessary foundation for the development of management practices for establishing national parks in Xinjiang, China.

## 2 Materials and Methods

### 2.1 Alien Woody Species Distribution Data

We collected data on 250 alien woody species from the China Invasive Species List (Lin, Xiao, and Ma 2022). The distribution data of alien woody species were downloaded from the Global Biodiversity Information Facility (GBIF; https://www.gbif.org/) database and the Chinese Virtual Herbarium (CVH; https://www.cvh.ac.cn/). Inaccurate coordinates associated with obvious latitude and longitude mismatches were corrected when possible before analysis. We downloaded species datasets globally and conducted predictions and risk assessments of these invasive species on a global scale. All extracted data were rasterized at a resolution of 10.0 arc‐minute cells (16.0 km at the equator) to minimize the effects of sampling bias and avoid georeferencing‐related errors, obvious misidentifications, and duplicate records in each grid cell (Jarnevich et al. 2015; Meyer, Weigelt, and Kreft 2016). Finally, more than 25 geographic data points were screened for model accuracy, and 250 alien woody species were selected and used as inputs for the species distribution model. State‐level nature reserve data were downloaded by searching for state‐level nature reserve boundary data in the Resource and Environmental Science and Data Center (https://www.resdc.cn/Default.aspx).

### 2.2 Climatic and Topographic Data

To obtain historical and future climate data, we downloaded 19 bioclimatic variables from historical (near current, 1970–2000) and predicted future (2080–2100) data, respectively, from the WorldClim website (www.worldclimm.org) at a resolution of 10 arc‐minutes (Hijmans et al. 2005). The predicted future data used in this study correspond to the latest IPCC‐CMIP6 model (Estoque et al. 2020). One of the main sets of simulations based on such models are future climate scenarios, in which models are given a common set of future concentrations of greenhouse gases and aerosols as well as other climate forcings to predict future climate conditions (Estoque et al. 2020). Climate scenarios constructed from the perspective of socio‐economic change, that is, the shared socio‐economic pathways (SSPs), promote comprehensive research on the scientific foundation of, impacts of, vulnerability to, risks of, adaptation to, and mitigation of climate change. SSP245 corresponds to the CMIP5 RCP4.5 scenario updated for CMIP6, representing a combination of moderate social vulnerability and moderate radiative forcing. SSP585 corresponds to the CMIP5 RCP8.5 scenario updated for CMIP6 and is the only shared socioeconomic pathway that can achieve an anthropogenic radiative forcing of 8.5 W/m^2^ in 2100 (Estoque et al. 2020). We modeled the distribution of alien woody species in China using SSP245 and SSP585 as future climate scenarios. We chose these scenarios because recent political and environmental events have raised projections for carbon emissions, such as the recent fires in the Brazilian Amazon rainforest and Australia and the failure of global climate agreements. We selected three global circulation models (i.e., MIROCC—ES2l, MIROC6, MRI‐ESM2‐0) to capture two representative concentration pathways (i.e., SSP245 and SSP585). Projected data from future climate scenarios were averaged based on the above models (i.e., MIROCC—ES2l, MIROC6, MRI‐ESM2‐0). We performed a correlation analysis to calculate the relationships between the 19 bioclimatic variables to minimize model overfitting. We then removed all available environmental variables with Pearson correlation coefficients > 0.7 to avoid multicollinearity effects in model parameter estimation (Elith et al. 2011). Finally, four environmental variables were selected to construct the species distribution model. The four environmental variables were bio1 (annual mean temperature), bio4 (temperature seasonality), bio12 (annual precipitation), and bio15 (precipitation seasonality).

Terrain data were obtained from EarthEnv (https://www.earthenv.org; Zhang, Wang, and Wan 2023). Five variables with a resolution of 10 km—namely elevation, roughness, terrain roughness index (TRI), vector ruggedness measure (VRM), and terrain position index (TPI)—were selected as terrain data factors. As reliable future projections for topographic factor indices were not available and inclusion of static variables in models alongside dynamic variables can improve model performance, we kept these variables static in our projections (Li et al. 2015).

### 2.3 Species Distribution Model

The maximum entropy model (i.e., Maxent) was used to predict the plant species distributions using only existing species distribution data and environmental variables (Phillips, Anderson, and Schapire 2006). The species distribution probability values generated by the resulting Maxent model ranged from 0 to 1 (Phillips, Anderson, and Schapire 2006; Merow, Smith, and Silander 2013). The alien woody species distribution data were randomly divided into training data (75%) and test data (25%; Merow, Smith, and Silander 2013). Each dataset of randomized training and test data was replicated four times, the maximum number of background points was set to 10,000, and the regularization multiplier was set to 2; all other parameters were set to default values. For model evaluation, we used the area under the curve (AUC) statistic, which accounts for the area under the receiver operating characteristic (ROC) curve, in which the sensitivity of habitat suitability corresponds to the rate of false positives for all possible habitat suitability values (Elith et al. 2011). An AUC value greater than 0.7 indicates that the model predictions are valid (Elith et al. 2011).

### 2.4 Data Analysis

Firstly, we used the average distribution probability of alien woody species from 11 current nature reserves in Xinjiang as a threshold to evaluate the level of invasion risk in three potential national parks in Xinjiang, as Xinjiang's nature reserves and potential national parks contain similar ecosystems (Sandvik 2023). We used statistical analysis methods to overlay three potential national parks and 11 nature reserves in Xinjiang with 250 distribution maps of exotic woody species and obtain the probability values and distribution maps of exotic woody species in the three potential national parks (Tianshan, Karamaili, and Altai Kanas) and Xinjiang's nature reserves.

Secondly, we used Kruskal–Wallis H‐test methods in SPSS version 26.0 (IBM Corp., Armonk, NY, USA) to compare the averages and differences in the distribution probabilities of alien woody species in the three potential national parks and the currently protected areas in Xinjiang under different climate scenarios. Under various climatic conditions, when the distribution probability of woody species inside and outside of potential national parks in Xinjiang was higher than that of invasive woody species in Xinjiang's nature reserves, then the probability of invasive woody species in the potential park was defined as high. If this distribution probability was lower than the mean distribution probability of invasive woody species in Xinjiang's nature reserves, then the probability of invasive woody species in the potential national park was defined as low.

Thirdly, we downloaded vegetation‐type data spanning China with a 1‐km resolution from the resource and environmental science data platform (http://www.resdc.cn/), overlayed the probability map of invasive woody plant distributions with the vegetation‐type data map of Xinjiang, and thus obtained the mean distribution probability of invasive woody species in the three potential national parks in Xinjiang across different ecosystems.

Finally, we used the fishing net analysis method and the average distribution of alien woody species in 11 nature reserves of Xinjiang as a threshold to calculate the distribution probability of each of the 250 invasive woody species in the three potential national parks and Xinjiang's nature reserves under different climate scenarios (Chi et al. 2017; Shrestha et al. 2021; Yang et al. 2023). Under the current and projected future climate scenarios, if the probability of invasion of a species in a potential national park in Xinjiang was greater than the average probability of the invasion distribution in Xinjiang's nature reserves by more than 10%, then the species was considered a high‐risk invasive woody plant species (Yang et al. 2023).

## 3 Results

### 3.1 Risk of Alien Woody Species Invasion in Potential National Parks in Xinjiang Under Climate Change

**FIGURE 1:**
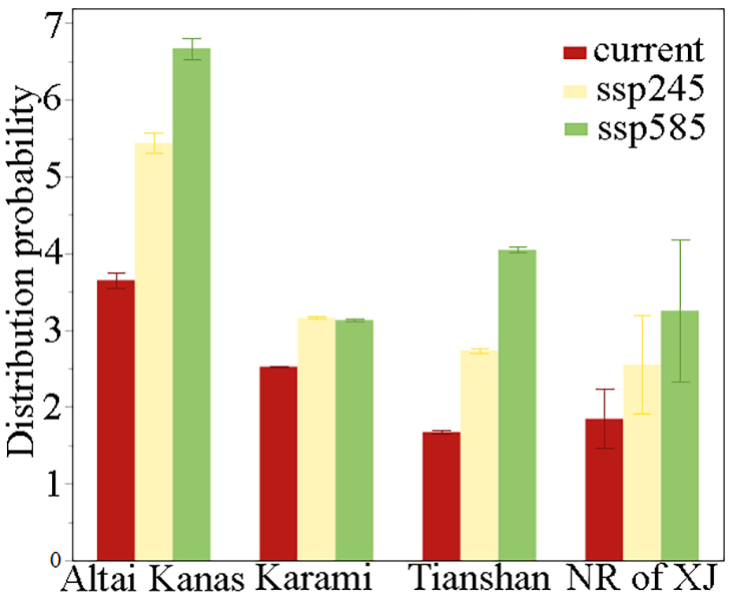
Under the current and future climate, the average distribution probability of alien woody species in the Tianshan, Altai Kanas, and Karamaili potential national parks and the current nature reserves of Xinjiang, China. NR of XJ, nature reserves in Xinjiang; ssp245, under the ssp245 climate scenario; ssp585, under the ssp585 climate scenario.

**FIGURE 2:**
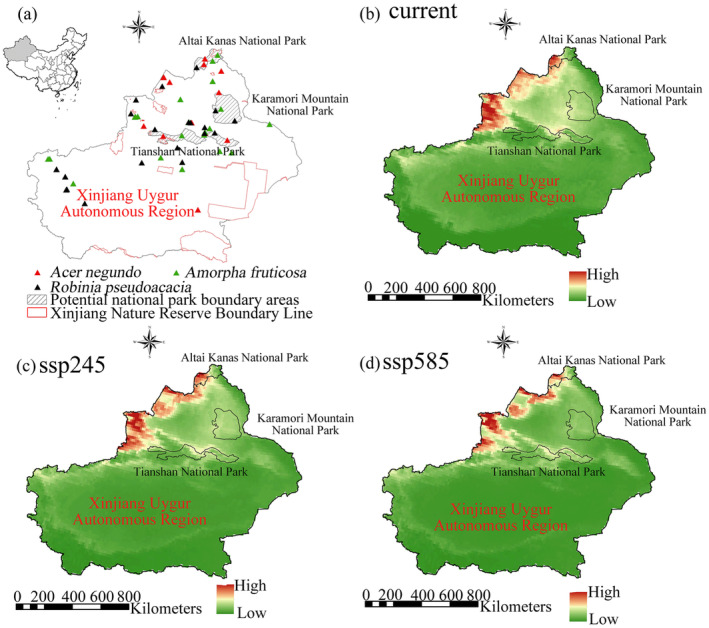
Distribution map of the entire Xinjiang Autonomous Region under different climate scenarios (including the distribution probability of alien woody species in three potential national parks and 11 nature reserves in Xinjiang). (a) Overview of the research area. (b) Probability map of the distribution of alien woody species under the current climate. (c) Probability map of the distribution of alien woody species under the ssp245 climate scenario. (d) Probability map of the distribution of alien woody species under the ssp585 climate scenario.

**FIGURE 3:**
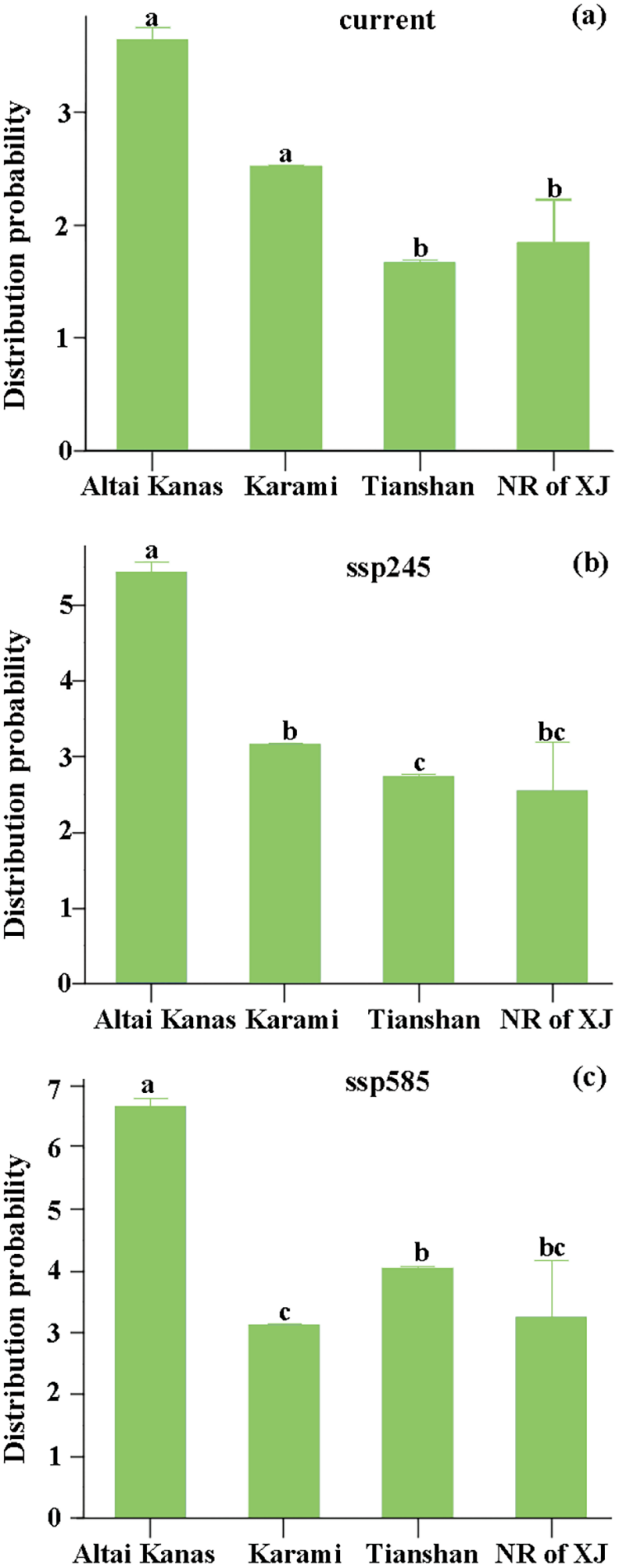
Comparison of the differences in the mean distribution probability of alien woody species in the Tianshan, Altai Kanas, and Karamaili potential national parks and the nature reserves of Xinjiang under the (a) current, (b) ssp245, and (c) ssp585 climate scenarios based on Kruskal–Wallis *H*‐tests. Bars labeled with different letters are significantly different at a threshold of *p* < 0.05.

For both training datasets, the AUC values of all species exceeded 0.9 (Table S1), indicating that our model performed well. Under the current climate conditions, the areas can be ranked in descending order of their distribution probabilities of invasive species as follows: Altai Kana, Karamaili, Xinjiang Nature Reserves, Tianshan. In the future climate scenario SSP245, the areas can be ranked in descending order of their distribution probabilities of invasive species as follows: Altai Kana, Karamaili, Tianshan, and Xinjiang nature reserves. In the future scenario SSP585, the areas can be ranked in descending order of their distribution probabilities of invasive species as follows: Altai Kana, Tianshan, Xinjiang nature reserves, Karamaili (Figure 1). Under climate change, the probability of the invasive plant distribution in Altai Kanas, Karamaili, and Tianshan potential national parks and Xinjiang nature reserves was determined to be gradually increasing (Figure 1; Table S2). The areas with a high probability of invasive species in Altai Kanas were mainly distributed in the southwest of the region (Figure 2). Under the current climate conditions, the probability of invasive species in Altai Kanas and Karamaili differed significantly from that of Xinjiang nature reserves (Figure 3). In the SSP245 and SSP585 scenarios, there was still a significant difference in the distribution probability of invasive plants between the Altai Kanas potential national park and Xinjiang nature reserves (Figure 3).

### 3.2 The Distribution Probability of Invasive Woody Species in Potential National Parks and Their Relationship With Ecosystems

**FIGURE 4:**
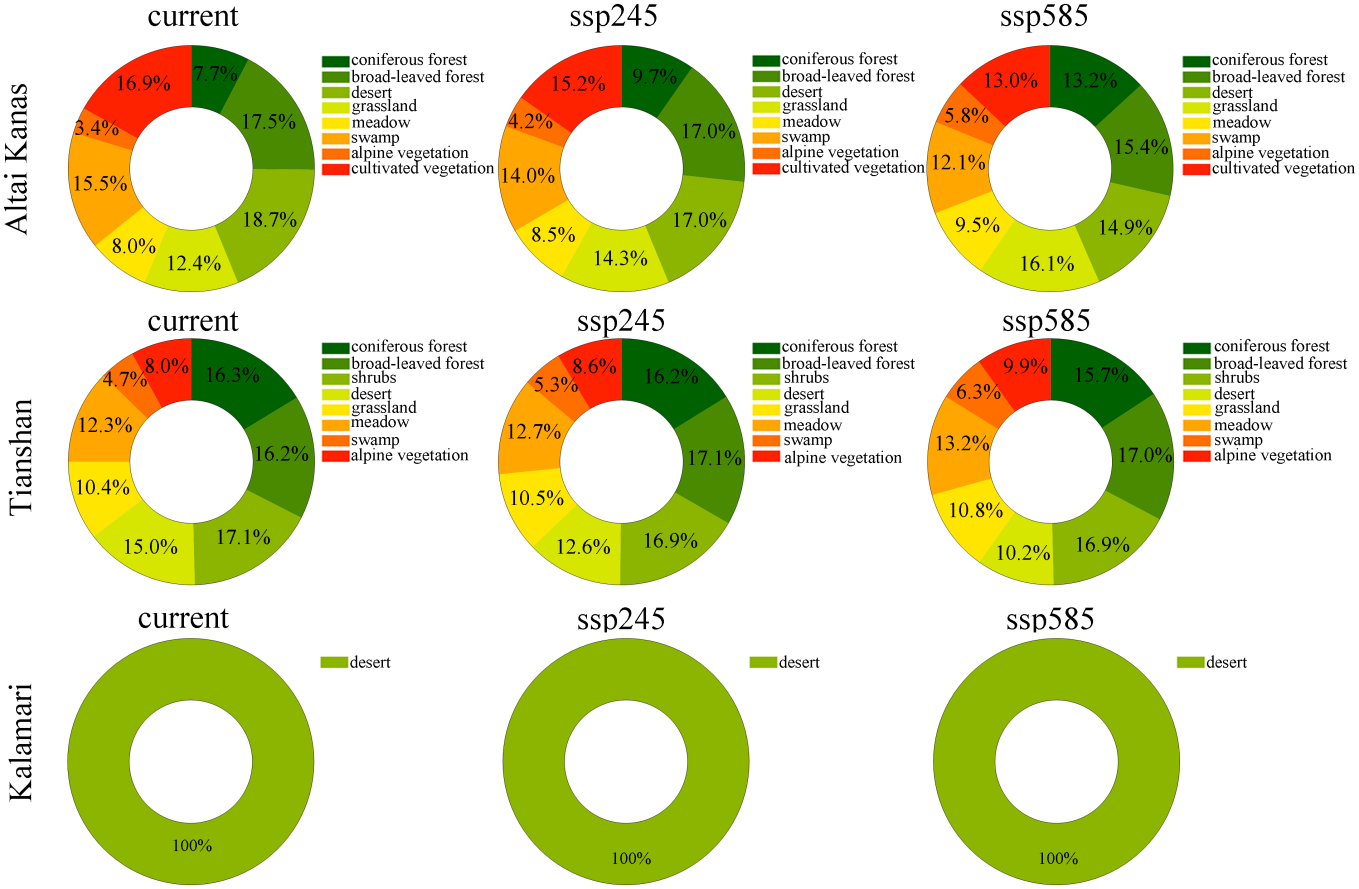
The distribution probability of alien woody species in different ecosystems in the Altai Kanas, Tianshan, and Karamaili potential national parks. ssp245, under the ssp245 climate scenario; ssp585, under the ssp585 climate scenario.

In Altai Kanas and Tianshan potential national parks, the probability of invasion into ecosystems with broad‐leaved forests, deserts, grasslands, swamps, and cultivated vegetation was relatively high, accounting for more than 15% (Figure 4). Under climate change, the distribution probability of invasive woody species increased for coniferous forest, alpine, and grassland vegetation types. In Tianshan, the probability of invasive woody species invading broad‐leaved forests, coniferous forests, shrublands, and desert vegetation types was relatively high, all reaching over 15% (Figure 4). However, under climate change, the proportion of invasive plants in broad‐leaved forests, alpine vegetation, swamps, meadows, and grassland vegetation types tended to increase, while the proportion of invasive plants in ecosystems with coniferous forests, deserts, and shrubs vegetation types was predicted to decrease with climate change. In the potential Karamaili National Park, the desert is the only major vegetation type, and the probability of invasive vegetation distribution in the desert was determined to be relatively high (Figure 4).

### 3.3 List of Alien Woody Species With High Invasion Risk in the Potential National Park Sites

The top 10% of species with a distribution probability higher than the threshold of Xinjiang nature reserves for alien woody species were defined as high‐risk species. Under current and future climate conditions, potential high‐risk species in the potential Altai National Park include *Acer negundo*, *Amorpha fruticosa*, *Betula pubescens*, *Fraxinus pennsylvanica*, *Populus deltoides*, *Populus nigra*, *Populus tremuloides*, and *Ulmus laevis*. The species that are not high risk in the current climate but will become high risk in the future include *Acer platanoides*, *Alnus glutinosa*, *Berberis vulgaris*, *Genista tinctoria*, *Lonicera xylosteum*, *Quercus robur*, *Ribes aureum*, *Salix nigra*, and *Tilia cordata* (Table 1). Under the current and future climate conditions, potential high‐risk species in the potential Tianshan national park include *Acer negundo*, *Pinus contorta*, and *Robinia pseudoacacia*; *Amorpha fruticosa*, *Pinus ponderosa*, *Populus deltoides*, *Ribes nigrum*, and *Sorbaria tomentosa* are currently not high‐risk species but were predicted to become high‐risk species for this area in the future. Under the current and future climate conditions, potential high‐risk species in the potential Karamaili National Park include *Amorpha fruticosa*, *Populus deltoides*, *Populus nigra*, and *Ribes nigrum*; *Fraxinus pennsylvanica* and *Ribes aureum* are currently not high‐risk species but were predicted to become high‐risk species in this area in the future (Table 1).

**TABLE 1:** With climate change, the distribution probability of woody invasive plants in three potential national parks in Xinjiang is gradually increasing.

Species	Altai kanas potential national park			Tianshan potential national park			Kalamari potential national park		
	Current	ssp245	ssp558	Current	ssp245	ssp558	Current	ssp245	ssp558
*Acer negundo*	+	+	+	+	+	+			
*Acer platanoides*			+						
*Alnus glutinosa*			+						
*Amorpha fruticosa*	+	+	+		+	+	+	+	+
*Berberis vulgaris*			+						
*Betula pubescens*	+	+	+						
*Fraxinus pennsylvanica*	+	+	+					+	+
*Genista tinctoria*		+	+						
*Lonicera xylosteum*			+						
*Pinus contorta*				+	+	+			
*Pinus ponderosa*					+	+			
*Populus balsamifera*									
*Populus deltoides*	+	+	+			+	+	+	+
*Populus nigra*	+	+	+				+	+	+
*Populus tremuloides*	+	+	+						
*Quercus robur*			+						
*Ribes aureum*		+	+						+
*Ribes nigrum*					+	+	+	+	+
*Robinia pseudoacacia*				+	+	+			
*Salix nigra*			+						
*Sorbaria tomentosa*						+			
*Tilia cordata*			+						
*Ulmus americana*									
*Ulmus laevis*	+	+	+						

*Note:* “+” represents species with a probability of invasive trees in potential national parks being 10% higher than the probability of invasive trees in Xinjiang protected areas.

## 4 Discussion

Our research indicates that potential national parks in Xinjiang are expected to be increasingly affected by invasive alien plants as a consequence of climate change. The potential Altai Kanas National Park has the highest probability of invasion risk among all three potential national parks in Xinjiang. This result may be owing to the fact that the potential Altai Kanas National Park is located at the northernmost point of Xinjiang, bordering Kazakhstan, Russia, and Mongolia. There are many international trade and transportation activities at the border, which may inadvertently introduce seeds or seedlings of foreign plant species. Meanwhile, border areas are often intertwined with different ecosystems, which may provide diverse niches for invasive species. Under projected climate change scenarios, the distribution probability of invasive plants in the Tianshan Mountains has also shown an increasing trend. The potential national park in the Tianshan Mountains is a biodiversity‐concentrated area mainly composed of the Tianshan Mountains. Owing to the altitudinal differences in this climate zone, the mountains cause a “wet island” effect in arid areas, creating diverse ecosystems and landscape types. The diversity of habitats can provide suitable conditions for various species (Chen et al. 2022).

Under current and future climate conditions, in order to successfully establish a national park in Xinjiang, we need to pay special attention to invasive species such as *Acer negundo*, *Ribes nigrum*, *Robinia pseudoacacia*, and *Amorpha fruticosa*, among other invasive plants. We also found that *Acer Negundo*, *Robinia pseudoacacia*, and *Amorpha fruticose* are currently distributed in three potential national parks and near national parks (Figure 2a). *Acer negundo* is an invasive tree species native to North America and one of the most aggressive invasive plants in forest stands (Bondarev 2013). This species is listed as a priority target species for research and control (Dgebuadze 2014). On a global scale, human disturbance of habitats and riverbanks in many parts of the Eurasian continent has led to its invasion into natural plant communities. In forest communities, the emergence of *Acer negundo* can hinder the growth of native plants, even hindering their renewal and disrupting the natural processes of succession (Abramova, Agishev, and Khaziakhmetov 2019). *Robinia pseudoacacia* is listed as one of the 40 most invasive woody angiosperms globally, and it is classified as highly invasive in several databases (e.g., EPPO, ISSG, DAISIE, CABI), and has also been identified as one of the 26 most harmful plants in Europe (Rumlerová et al. 2016; Sádlo et al. 2017). This species is native to the southeastern part of North America. It was introduced outside of its native range in the late 19th century to stabilize riverbanks and prevent severe erosion (Nilsen and Huebner 2023). *Robinia pseudoacacia* tends to be found in areas with loose soil, periodic flooding, and partial shading. It appears in large numbers on abandoned farmland and pastures. In the past 30–40 years, owing to the transformation of land use from arable land to artificial forests in the Tisza floodplain as well as poor forest management, the spread of *Robinia pseudoacacia* has been exceptionally rapid (Nilsen and Huebner 2023). With the increase of climate warming, changes in precipitation patterns, and extreme weather events, invasive plants have gained more opportunities for growth and diffusion, which may lead to further losses of biodiversity, affect ecosystem services, and even threaten agriculture and human well‐being.

The potential area of Tianshan National Park is mainly composed of major ecosystems such as forests, grasslands, and swamps, which together contain the biodiversity of the Tianshan region. Among them, deserts, grasslands, and forests account for 50.4%, 21.3%, and 5.2% of its area, respectively. The main ecosystem type in the potential area of Altay Mount Taishan National Park is grasslands, accounting for about 44% of its area; the percentage of area covered by desert ecosystems is about 25%, while forest ecosystems cover about 23%. The potential Karamaili National Park includes the main types of desert ecosystems, and the area of natural desert ecosystems accounts for more than 99% of the total area of the potential Karamaili National Park. There are also differences in the invasion distribution probability between different habitats and vegetation types. Our research indicates that the distribution probability of invasive plants in specific types of ecosystems of the potential national parks, such as coniferous forests, broad‐leaved forests, deserts, and swamps, is high. This result is consistent with previous research (Schirmel et al. 2016). Research has shown that broad‐leaved forests provide diverse habitat conditions, including spatial variation in lighting, temperature, humidity, and soil conditions, which may be beneficial for the adaptation and growth of various invasive plants (van Hengstum et al. 2014). Invasive plants can utilize new ecological niches, especially at the edges or in the damaged areas of native forests. There are also studies indicating that swamp ecosystems are particularly susceptible to invasion by foreign plant species. This is because swamp soils are usually nutrient‐poor, and local plants may face significant survival pressures, providing niche opportunities for invasive plants (Zhang et al. 2017). At the same time, swamps have unique hydrological and soil conditions, which may provide space and resources for certain invasive plants.

The management of invasive alien woody species in forests often results in an increase in the coverage and diversity of local plants. Such management can improve the establishment and vigor of local tree species. Although the response measures vary, the invaded areas may require strengthened management to achieve the intended results. Despite extensive efforts by the scientific community and research organizations to improve our understanding of the ecological impacts of introduced species in national parks, our findings suggest that in the face of climate change, more action is needed to minimize the invasion capacity of introduced species in order to maintain the core functions of ecosystems through protecting local biodiversity within protected areas.

WorldClim is a reliable data source that provides ready‐made information for determining baselines and predicting future scenarios (Poggio, Simonetti, and Gimona 2018). One important application is bioclimate modeling, which is used to study the climate‐associated range changes of local species, invasive plants, and pest species (Graham et al. 2004). This type of change is typically modeled using statistical methods developed for climate data and projected climate scenarios. Climate data are widely used to examine and predict the areas that species may occupy on multiple spatial and temporal scales (Carnaval et al. 2009). Though WorldClim is commonly used for species modeling in different cases, regions, and applications (Curtis and Bradley 2016), it has some key limitations: WorldClim lacks the spatial details typically required for impact assessment models, limiting its use in predicting spatial heterogeneity (Daly, Neilson, and Phillips 1994). There are more difficult issues associated with steep mountainous terrain, where medium‐resolution grid elements can span climate environments with altitude differences of several hundred meters. In addition, our research has three limitations: (1) Our study did not consider the impact of microclimate conditions on the potential national parks. We simulated the distribution of invasive woody species in potential national parks in Xinjiang without accounting for this level of heterogeneity. Future research should comprehensively consider factors such as climate conditions in small‐scale regions to accurately assess the potential distribution of invasive woody species in potential national parks and other conservation areas. (2) We did not examine the correlation between climate variables and altitude, as altitude is usually negatively correlated with temperature because as altitude increases, temperature typically decreases. If variables are highly correlated in the model, it may lead to multicollinearity issues, affecting the stability of the model and the accuracy of predictions. Therefore, in future research, the correlation between altitude and climate should also be considered. (3) LDD is an important driver of ecological and evolutionary patterns, and dispersal ability is one of the key factors affecting species distribution. However, we did not incorporate the dispersal ability of alien woody into our model, which may lead to an insufficient risk assessment of alien woody, inaccurate predictions, and improper management measures. In future research, the impact of dispersal ability on the distribution of alien woody should be considered in the model.

## 5 Conclusion

Plant invasion prevention and control measures can be promoted by assessing the risk of alien woody species invasion in potential national park sites in China. Under the current and projected future climate conditions, particular attention should be given to the invasion potential and spread of *Acer negundo*, *Ribes nigrum*, *Robinia pseudo acacia*, and *Amorpha fruticose* in order to effectively prevent their invasion and control the risk of ecosystem disturbances by these invasive alien woody species. Our study provides new insights into the long‐term biodiversity conservation practices of national parks in Xinjiang, China.

## Author Contributions

**Fei‐Xue Zhang:** resources (equal), software (equal), visualization (equal), writing – original draft (equal). **Hong‐Li Li:** investigation (equal), project administration (equal), supervision (equal), writing – review and editing (equal). **Ji‐Zhong Wan:** project administration (equal), validation (equal), writing – review and editing (equal).

## Ethics Statement

This article does not contain any studies with human participants or animals performed by any of the authors.

## Conflicts of Interest

The authors declare no conflicts of interest, and the manuscript was approved by all authors for publication.

## Supporting information


Tables S1‐S2.

## Data Availability

We used open‐access data from the Global Biodiversity Information Facility database (GBIF, https://www.gbif.org/), WorldClim (http://worldclim.org), and EarthEnv project (https://www.earthenv.org).
