# Supplementary material for: An Invasion Risk Assessment of Alien Woody Species in Potential National Park Sites in Xinjiang, China, Under Climate Change
Source: Ecol Evol. 2024 Oct 8;14(10):e70394. doi: 10.1002/ece3.70394 (PMC11459206; doi:10.1002/ece3.70394)
Supplement: Supplementary file 1 — Tables S1‐S2. [file ECE3-14-e70394-s001.docx]

**Supporting materials**

**Table S1** Contribution rates of AUC and environmental variables of 250 alien woody species.

| Species | Training AUC | Test AUC | bio1 | bio12 | bio15 | bio4 |
| --- | --- | --- | --- | --- | --- | --- |
| *Cryptomeria japonica* | 0.914 | 0.912 | 46.999 | 44.807 | 0.202 | 6.562 |
| *Chamaecyparis lawsoniana* | 0.890 | 0.890 | 45.188 | 24.363 | 5.944 | 21.989 |
| *Cupressus sempervirens* | 0.897 | 0.896 | 59.151 | 9.370 | 0.731 | 29.378 |
| *Juniperus occidentalis* | 0.967 | 0.966 | 30.961 | 5.953 | 0.767 | 29.781 |
| *Juniperus virginiana* | 0.837 | 0.837 | 20.213 | 46.599 | 5.111 | 26.752 |
| *Cedrus deodara* | 0.913 | 0.912 | 47.652 | 25.016 | 4.508 | 22.220 |
| *Abies alba* | 0.844 | 0.844 | 38.434 | 24.909 | 5.047 | 31.264 |
| *Abies amabilis* | 0.975 | 0.974 | 31.802 | 42.162 | 1.772 | 14.937 |
| *Abies grandis* | 0.906 | 0.906 | 48.545 | 18.118 | 0.700 | 31.753 |
| *Abies nordmanniana* | 0.948 | 0.947 | 33.331 | 18.245 | 23.350 | 24.689 |
| *Abies procera* | 0.960 | 0.959 | 41.902 | 18.306 | 0.389 | 36.012 |
| *Abies sachalinensis* | 0.980 | 0.978 | 17.726 | 45.971 | 5.223 | 18.856 |
| *Pseudotsuga menziesii* | 0.788 | 0.788 | 53.473 | 8.007 | 1.456 | 35.836 |
| *Larix decidua* | 0.815 | 0.815 | 43.587 | 30.310 | 5.721 | 20.117 |
| *Larix kaempferi* | 0.910 | 0.909 | 55.953 | 37.690 | 0.996 | 4.341 |
| *Picea abies* | 0.708 | 0.708 | 44.829 | 35.981 | 8.935 | 9.998 |
| *Picea sitchensis* | 0.887 | 0.887 | 34.916 | 24.370 | 1.522 | 36.129 |
| *Pinus banksiana* | 0.905 | 0.904 | 41.632 | 36.773 | 1.867 | 14.710 |
| *Pinus canariensis* | 0.970 | 0.967 | 53.301 | 8.052 | 3.282 | 23.932 |
| *Pinus caribaea* | 0.959 | 0.955 | 8.896 | 56.088 | 9.406 | 20.590 |
| *Pinus contorta* | 0.851 | 0.851 | 54.956 | 11.484 | 1.224 | 30.632 |
| *Pinus elliottii* | 0.968 | 0.967 | 13.798 | 46.047 | 4.323 | 15.352 |
| *Pinus glabra* | 0.987 | 0.986 | 7.517 | 48.941 | 2.348 | 23.103 |
| *Pinus halepensis* | 0.905 | 0.905 | 61.781 | 6.566 | 0.917 | 29.476 |
| *Pinus luchuensis* | 0.987 | 0.984 | 7.516 | 58.414 | 2.572 | 20.813 |
| *Pinus monticola* | 0.959 | 0.958 | 34.737 | 14.861 | 3.275 | 28.933 |
| *Pinus nigra* | 0.841 | 0.841 | 51.780 | 14.394 | 12.307 | 21.334 |
| *Pinus oocarpa* | 0.966 | 0.964 | 1.770 | 13.049 | 14.666 | 36.044 |
| *Pinus parviflora* | 0.977 | 0.976 | 17.831 | 54.340 | 0.429 | 26.827 |
| *Pinus patula* | 0.958 | 0.957 | 30.088 | 19.808 | 0.175 | 33.439 |
| *Pinus pinaster* | 0.888 | 0.888 | 57.180 | 8.462 | 1.841 | 31.950 |
| *Pinus pinea* | 0.927 | 0.926 | 60.274 | 11.468 | 0.857 | 26.272 |
| *Pinus ponderosa* | 0.897 | 0.897 | 47.896 | 6.801 | 0.554 | 16.795 |
| *Pinus radiata* | 0.923 | 0.923 | 41.828 | 5.895 | 0.994 | 48.945 |
| *Pinus rigida* | 0.952 | 0.951 | 28.008 | 40.556 | 14.190 | 16.434 |
| *Pinus strobus* | 0.808 | 0.808 | 39.790 | 48.622 | 6.926 | 4.178 |
| *Pinus taeda* | 0.940 | 0.940 | 14.336 | 53.281 | 9.117 | 21.578 |
| *Pinus thunbergii* | 0.966 | 0.965 | 28.834 | 41.378 | 0.485 | 16.148 |
| *Annona squamosa* | 0.898 | 0.896 | 37.869 | 30.484 | 1.849 | 24.257 |
| *Persea americana* | 0.865 | 0.864 | 18.465 | 13.921 | 0.727 | 59.053 |
| *Cinnamomum verum* | 0.927 | 0.922 | 13.656 | 68.142 | 1.540 | 10.747 |
| *Arenga pinnata* | 0.953 | 0.942 | 8.500 | 77.298 | 1.487 | 0.984 |
| *Borassus flabellifer* | 0.967 | 0.962 | 60.103 | 22.006 | 5.948 | 3.781 |
| *Washingtonia filifera* | 0.940 | 0.936 | 65.043 | 12.611 | 2.248 | 6.646 |
| *Washingtonia robusta* | 0.932 | 0.928 | 61.860 | 5.938 | 1.802 | 13.393 |
| *Phytelephas macrocarpa* | 0.974 | 0.969 | 0.570 | 37.264 | 6.734 | 52.026 |
| *Roystonea oleracea* | 0.969 | 0.965 | 3.815 | 35.552 | 7.592 | 46.637 |
| *Elaeis guineensis* | 0.936 | 0.935 | 16.418 | 28.578 | 2.207 | 49.161 |
| *Archontophoenix cunninghamiana* | 0.979 | 0.977 | 30.243 | 31.562 | 7.629 | 25.086 |
| *Ptychosperma elegans* | 0.975 | 0.967 | 23.138 | 27.973 | 7.716 | 15.592 |
| *Ptychosperma macarthurii* | 0.953 | 0.949 | 9.282 | 45.798 | 0.031 | 19.235 |
| *Berberis darwinii* | 0.958 | 0.958 | 38.569 | 18.759 | 0.549 | 38.978 |
| *Berberis microphylla* | 0.981 | 0.980 | 36.640 | 0.747 | 0.761 | 58.385 |
| *Berberis thunbergii* | 0.801 | 0.800 | 54.095 | 34.623 | 5.228 | 5.543 |
| *Berberis vulgaris* | 0.802 | 0.801 | 60.348 | 24.266 | 8.044 | 7.241 |
| *Hakea salicifolia* | 0.977 | 0.976 | 35.312 | 13.840 | 6.444 | 42.471 |
| *Grevillea banksii* | 0.969 | 0.966 | 37.666 | 25.072 | 3.886 | 21.392 |
| *Grevillea robusta* | 0.880 | 0.878 | 58.689 | 25.330 | 0.932 | 14.144 |
| *Dillenia suffruticosa* | 0.982 | 0.980 | 1.316 | 54.708 | 4.286 | 31.661 |
| *Ribes alpinum* | 0.823 | 0.823 | 43.835 | 28.836 | 5.546 | 21.311 |
| *Ribes aureum* | 0.827 | 0.827 | 82.677 | 8.165 | 2.807 | 5.687 |
| *Ribes multiflorum* | 0.980 | 0.958 | 31.975 | 19.067 | 3.673 | 12.685 |
| *Ribes nigrum* | 0.753 | 0.752 | 63.297 | 28.876 | 3.400 | 1.489 |
| *Ribes uva-crispa* | 0.769 | 0.769 | 56.014 | 31.169 | 7.121 | 5.445 |
| *Copaifera officinalis* | 0.962 | 0.943 | 8.217 | 10.335 | 1.575 | 68.858 |
| *Tamarindus indica* | 0.863 | 0.862 | 46.732 | 12.213 | 2.898 | 36.131 |
| *Cassia fistula* | 0.887 | 0.884 | 49.172 | 32.286 | 1.773 | 10.825 |
| *Senna multijuga* | 0.931 | 0.928 | 4.072 | 59.119 | 4.006 | 30.180 |
| *Senna siamea* | 0.888 | 0.887 | 19.784 | 23.995 | 2.471 | 51.533 |
| *Senna spectabilis* | 0.911 | 0.910 | 13.952 | 14.704 | 1.375 | 62.937 |
| *Schizolobium parahyba* | 0.942 | 0.938 | 6.391 | 57.009 | 3.766 | 30.378 |
| *Delonix regia* | 0.854 | 0.852 | 41.605 | 14.951 | 1.905 | 37.508 |
| *Mimosa pigra* | 0.852 | 0.850 | 20.031 | 19.114 | 0.434 | 58.648 |
| *Acaciella glauca* | 0.938 | 0.924 | 21.739 | 13.955 | 4.850 | 40.778 |
| *Albizia lebbeck* | 0.883 | 0.881 | 59.204 | 25.862 | 2.245 | 9.197 |
| *Acacia adunca* | 0.995 | 0.993 | 36.559 | 17.668 | 17.171 | 25.051 |
| *Acacia decurrens* | 0.958 | 0.957 | 47.526 | 12.801 | 5.788 | 32.875 |
| *Acacia elata* | 0.988 | 0.987 | 36.412 | 13.616 | 4.086 | 43.810 |
| *Acacia fimbriata* | 0.980 | 0.979 | 20.991 | 26.589 | 13.680 | 37.529 |
| *Acacia holosericea* | 0.949 | 0.948 | 40.942 | 16.174 | 26.724 | 10.211 |
| *Acacia implexa* | 0.952 | 0.951 | 30.683 | 18.453 | 11.491 | 38.801 |
| *Acacia iteaphylla* | 0.983 | 0.982 | 43.490 | 6.836 | 0.545 | 36.937 |
| *Acacia mangium* | 0.943 | 0.940 | 13.370 | 63.722 | 0.916 | 12.179 |
| *Acacia melanoxylon* | 0.897 | 0.897 | 36.089 | 14.898 | 1.942 | 46.627 |
| *Acacia paradoxa* | 0.956 | 0.956 | 50.387 | 7.581 | 2.877 | 36.175 |
| *Acacia podalyriifolia* | 0.967 | 0.965 | 49.090 | 14.388 | 2.807 | 29.218 |
| *Myroxylon balsamum* | 0.941 | 0.934 | 3.449 | 13.982 | 1.819 | 78.256 |
| *Styphnolobium japonicum* | 0.933 | 0.931 | 66.628 | 18.606 | 0.078 | 12.140 |
| *Cytisus nigricans* | 0.960 | 0.959 | 46.682 | 20.121 | 4.532 | 27.301 |
| *Cytisus scoparius* | 0.768 | 0.767 | 49.558 | 20.395 | 1.258 | 28.131 |
| *Chamaecytisus ratisbonensis* | 0.985 | 0.983 | 45.565 | 14.734 | 3.947 | 29.949 |
| *Spartium junceum* | 0.878 | 0.877 | 62.145 | 6.844 | 0.064 | 30.313 |
| *Genista germanica* | 0.928 | 0.928 | 32.541 | 20.484 | 16.912 | 29.581 |
| *Genista pilosa* | 0.901 | 0.901 | 32.698 | 16.709 | 23.925 | 26.653 |
| *Genista sagittalis* | 0.929 | 0.929 | 26.803 | 17.326 | 29.664 | 24.067 |
| *Genista tinctoria* | 0.812 | 0.812 | 57.932 | 25.224 | 12.504 | 3.623 |
| *Ulex europaeus* | 0.827 | 0.827 | 39.239 | 20.978 | 3.108 | 35.307 |
| *Amorpha fruticosa* | 0.820 | 0.820 | 48.130 | 23.221 | 1.486 | 26.795 |
| *Dalbergia sissoo* | 0.926 | 0.920 | 57.057 | 16.211 | 7.983 | 15.126 |
| *Tipuana tipu* | 0.952 | 0.950 | 56.921 | 7.112 | 2.074 | 28.526 |
| *Tephrosia candida* | 0.961 | 0.952 | 14.920 | 76.067 | 2.061 | 3.714 |
| *Erythrina poeppigiana* | 0.956 | 0.953 | 4.544 | 16.590 | 2.567 | 68.371 |
| *Hippocrepis emerus* | 0.912 | 0.912 | 29.180 | 19.969 | 3.236 | 46.982 |
| *Gliricidia sepium* | 0.904 | 0.902 | 9.186 | 21.714 | 2.454 | 61.782 |
| *Robinia pseudoacacia* | 0.723 | 0.722 | 71.543 | 20.452 | 2.889 | 4.925 |
| *Medicago arborea* | 0.965 | 0.964 | 51.095 | 9.126 | 0.602 | 26.785 |
| *Polygala myrtifolia* | 0.939 | 0.939 | 47.105 | 4.629 | 1.663 | 38.216 |
| *Rubus moluccanus* | 0.948 | 0.946 | 11.211 | 50.158 | 14.185 | 16.721 |
| *Rosa pendulina* | 0.922 | 0.921 | 29.425 | 24.162 | 12.325 | 29.951 |
| *Physocarpus opulifolius* | 0.845 | 0.845 | 45.690 | 34.620 | 8.220 | 10.299 |
| *Prunus domestica* | 0.796 | 0.796 | 58.584 | 23.334 | 5.201 | 12.476 |
| *Sorbaria tomentosa* | 0.977 | 0.971 | 35.535 | 15.540 | 2.726 | 17.699 |
| *Spiraea alba* | 0.865 | 0.865 | 46.442 | 43.045 | 3.026 | 4.985 |
| *Spiraea douglasii* | 0.902 | 0.901 | 48.422 | 22.876 | 0.432 | 26.525 |
| *Pyracantha coccinea* | 0.848 | 0.848 | 55.615 | 15.625 | 6.593 | 21.637 |
| *Amelanchier lamarckii* | 0.947 | 0.946 | 37.270 | 15.369 | 23.135 | 18.871 |
| *Amelanchier ovalis* | 0.903 | 0.903 | 24.837 | 11.434 | 16.705 | 41.157 |
| *Cotoneaster simonsii* | 0.958 | 0.957 | 32.279 | 19.782 | 1.747 | 40.193 |
| *Rhamnus alaternus* | 0.893 | 0.893 | 54.816 | 8.798 | 0.732 | 34.616 |
| *Ulmus americana* | 0.873 | 0.873 | 17.648 | 43.639 | 4.232 | 32.068 |
| *Ulmus laevis* | 0.866 | 0.866 | 55.910 | 19.268 | 15.669 | 5.244 |
| *Ulmus minor* | 0.802 | 0.802 | 62.292 | 15.298 | 4.465 | 17.551 |
| *Artocarpus altilis* | 0.923 | 0.922 | 8.155 | 35.797 | 1.825 | 46.426 |
| *Artocarpus heterophyllus* | 0.900 | 0.897 | 24.881 | 65.688 | 0.630 | 5.156 |
| *Ficus benghalensis* | 0.922 | 0.920 | 56.557 | 22.403 | 4.703 | 6.962 |
| *Ficus religiosa* | 0.916 | 0.912 | 53.503 | 22.474 | 5.055 | 8.196 |
| *Ficus rubiginosa* | 0.950 | 0.949 | 29.970 | 23.114 | 3.519 | 38.434 |
| *Cecropia peltata* | 0.933 | 0.931 | 1.845 | 33.620 | 5.535 | 55.099 |
| *Fagus sylvatica* | 0.757 | 0.757 | 45.705 | 25.222 | 3.966 | 24.772 |
| *Castanea crenata* | 0.959 | 0.958 | 12.695 | 58.089 | 1.279 | 27.613 |
| *Castanea sativa* | 0.808 | 0.808 | 45.901 | 21.688 | 3.434 | 28.733 |
| *Quercus cerris* | 0.904 | 0.904 | 51.347 | 20.697 | 8.876 | 18.520 |
| *Quercus coccifera* | 0.914 | 0.913 | 55.596 | 11.065 | 0.760 | 31.633 |
| *Quercus petraea* | 0.814 | 0.814 | 44.316 | 23.989 | 5.498 | 26.091 |
| *Quercus rubra* | 0.772 | 0.772 | 48.817 | 38.924 | 7.408 | 4.406 |
| *Myrica gale* | 0.806 | 0.806 | 48.692 | 38.477 | 2.679 | 1.540 |
| *Juglans ailanthifolia* | 0.971 | 0.970 | 33.625 | 53.984 | 3.359 | 7.007 |
| *Juglans cinerea* | 0.936 | 0.936 | 17.629 | 38.379 | 19.311 | 24.254 |
| *Juglans nigra* | 0.844 | 0.844 | 31.902 | 38.636 | 9.092 | 19.730 |
| *Casuarina cunninghamiana* | 0.922 | 0.920 | 33.594 | 24.395 | 3.563 | 37.224 |
| *Casuarina equisetifolia* | 0.895 | 0.894 | 30.487 | 11.916 | 0.273 | 23.174 |
| *Casuarina glauca* | 0.982 | 0.976 | 21.357 | 21.725 | 3.528 | 26.560 |
| *Allocasuarina littoralis* | 0.949 | 0.948 | 11.401 | 31.525 | 8.302 | 47.355 |
| *Alnus glutinosa* | 0.715 | 0.715 | 56.721 | 31.846 | 7.219 | 3.522 |
| *Alnus rubra* | 0.961 | 0.960 | 37.535 | 39.328 | 4.123 | 17.358 |
| *Betula pubescens* | 0.702 | 0.701 | 55.025 | 34.080 | 6.610 | 3.162 |
| *Carpinus betulus* | 0.793 | 0.793 | 53.548 | 24.207 | 5.144 | 17.017 |
| *Averrhoa carambola* | 0.913 | 0.910 | 21.556 | 68.438 | 0.884 | 1.899 |
| *Pentadesma butyracea* | 0.976 | 0.973 | 15.688 | 31.861 | 8.500 | 40.902 |
| *Mammea americana* | 0.953 | 0.944 | 8.410 | 20.559 | 2.827 | 58.909 |
| *Galphimia glauca* | 0.955 | 0.951 | 12.265 | 11.625 | 12.451 | 35.794 |
| *Dovyalis caffra* | 0.968 | 0.967 | 30.942 | 11.154 | 2.410 | 44.842 |
| *Populus balsamifera* | 0.828 | 0.827 | 69.723 | 19.900 | 5.477 | 1.854 |
| *Populus deltoides* | 0.843 | 0.842 | 62.436 | 14.743 | 4.599 | 15.687 |
| *Populus nigra* | 0.778 | 0.777 | 70.785 | 16.426 | 5.006 | 7.278 |
| *Populus tremuloides* | 0.801 | 0.800 | 79.406 | 10.481 | 3.579 | 3.777 |
| *Salix nigra* | 0.894 | 0.893 | 23.868 | 41.841 | 3.601 | 28.580 |
| *Salix repens* | 0.822 | 0.822 | 39.525 | 29.759 | 10.161 | 18.749 |
| *Hevea brasiliensis* | 0.930 | 0.926 | 5.363 | 66.464 | 1.419 | 25.154 |
| *Aleurites moluccanus* | 0.936 | 0.934 | 22.117 | 62.796 | 1.592 | 3.234 |
| *Reutealis trisperma* | 0.965 | 0.937 | 6.110 | 36.962 | 3.530 | 40.550 |
| *Garcia nutans* | 0.966 | 0.947 | 13.838 | 42.251 | 4.951 | 29.213 |
| *Hura crepitans* | 0.933 | 0.930 | 3.641 | 20.375 | 3.393 | 69.177 |
| *Melianthus major* | 0.975 | 0.974 | 44.797 | 3.788 | 1.004 | 42.283 |
| *Sonneratia apetala* | 0.991 | 0.988 | 14.405 | 24.813 | 16.401 | 10.934 |
| *Callistemon linearis* | 0.976 | 0.972 | 36.493 | 19.588 | 11.193 | 31.069 |
| *Callistemon rigidus* | 0.944 | 0.922 | 50.816 | 18.230 | 0.873 | 10.711 |
| *Callistemon viminalis* | 0.929 | 0.927 | 44.507 | 20.176 | 3.029 | 28.146 |
| *Melaleuca hypericifolia* | 0.987 | 0.982 | 31.058 | 15.533 | 6.444 | 37.603 |
| *Corymbia citriodora* | 0.945 | 0.943 | 41.616 | 22.685 | 2.175 | 27.521 |
| *Corymbia maculata* | 0.973 | 0.971 | 41.327 | 14.288 | 4.059 | 32.679 |
| *Corymbia torelliana* | 0.965 | 0.960 | 31.682 | 31.132 | 7.360 | 16.825 |
| *Eucalyptus benthamii* | 0.978 | 0.972 | 11.259 | 3.111 | 40.913 | 43.339 |
| *Eucalyptus botryoides* | 0.979 | 0.978 | 39.370 | 12.421 | 7.312 | 33.625 |
| *Eucalyptus brassiana* | 0.982 | 0.974 | 16.222 | 40.446 | 18.310 | 12.170 |
| *Eucalyptus camaldulensis* | 0.869 | 0.867 | 46.309 | 14.965 | 2.334 | 32.704 |
| *Eucalyptus cinerea* | 0.966 | 0.964 | 50.157 | 14.163 | 2.165 | 30.945 |
| *Eucalyptus cladocalyx* | 0.976 | 0.975 | 46.607 | 7.743 | 0.366 | 34.435 |
| *Eucalyptus cloeziana* | 0.990 | 0.988 | 39.753 | 23.571 | 5.888 | 21.346 |
| *Eucalyptus diversicolor* | 0.993 | 0.991 | 33.397 | 6.097 | 3.551 | 40.430 |
| *Eucalyptus globulus* | 0.904 | 0.903 | 51.481 | 7.950 | 0.226 | 38.032 |
| *Eucalyptus gomphocephala* | 0.985 | 0.979 | 44.160 | 7.243 | 2.211 | 26.779 |
| *Eucalyptus grandis* | 0.974 | 0.967 | 38.081 | 20.295 | 4.505 | 30.167 |
| *Eucalyptus gunnii* | 0.983 | 0.979 | 29.104 | 16.469 | 14.478 | 38.985 |
| *Eucalyptus lehmannii* | 0.994 | 0.993 | 36.406 | 6.377 | 0.259 | 42.087 |
| *Eucalyptus megacornuta* | 0.985 | 0.972 | 40.086 | 12.131 | 0.030 | 29.687 |
| *Eucalyptus nitens* | 0.992 | 0.988 | 28.530 | 13.573 | 9.207 | 33.349 |
| *Eucalyptus pellita* | 0.963 | 0.953 | 4.741 | 56.102 | 7.428 | 20.406 |
| *Eucalyptus robusta* | 0.951 | 0.948 | 47.113 | 34.145 | 1.620 | 4.710 |
| *Eucalyptus saligna* | 0.974 | 0.973 | 34.653 | 28.405 | 8.527 | 25.681 |
| *Eucalyptus sideroxylon* | 0.965 | 0.964 | 54.896 | 11.499 | 4.740 | 27.765 |
| *Eucalyptus tereticornis* | 0.929 | 0.928 | 37.565 | 25.483 | 3.621 | 31.943 |
| *Eucalyptus urophylla* | 0.968 | 0.956 | 5.632 | 44.796 | 2.895 | 35.733 |
| *Eucalyptus viminalis* | 0.945 | 0.945 | 40.605 | 9.418 | 3.729 | 45.507 |
| *Metrosideros excelsa* | 0.982 | 0.981 | 28.491 | 10.277 | 1.188 | 40.644 |
| *Syzygium malaccense* | 0.942 | 0.940 | 2.899 | 35.980 | 2.889 | 53.274 |
| *Syzygium paniculatum* | 0.975 | 0.973 | 37.477 | 21.388 | 3.239 | 33.372 |
| *Psidium guineense* | 0.910 | 0.908 | 10.674 | 36.908 | 2.918 | 46.526 |
| *Pimenta dioica* | 0.950 | 0.945 | 15.171 | 51.564 | 8.684 | 21.054 |
| *Heterotis rotundifolia* | 0.949 | 0.946 | 6.718 | 47.460 | 3.325 | 40.562 |
| *Staphylea pinnata* | 0.926 | 0.926 | 45.181 | 19.543 | 16.669 | 17.897 |
| *Schinus molle* | 0.883 | 0.882 | 69.629 | 1.721 | 0.142 | 27.950 |
| *Acer negundo* | 0.697 | 0.697 | 68.433 | 19.642 | 4.096 | 7.371 |
| *Acer platanoides* | 0.723 | 0.722 | 57.754 | 31.498 | 9.144 | 1.059 |
| *Acer pseudoplatanus* | 0.754 | 0.754 | 44.698 | 26.140 | 4.736 | 24.354 |
| *Aesculus hippocastanum* | 0.768 | 0.768 | 61.947 | 23.757 | 6.363 | 7.530 |
| *Melicoccus bijugatus* | 0.955 | 0.953 | 4.351 | 17.798 | 7.564 | 60.132 |
| *Cedrela odorata* | 0.888 | 0.886 | 6.515 | 20.048 | 0.956 | 68.805 |
| *Azadirachta indica* | 0.859 | 0.857 | 68.219 | 21.189 | 6.492 | 2.427 |
| *Triumfetta bogotensis* | 0.954 | 0.949 | 3.733 | 21.869 | 2.317 | 56.349 |
| *Tilia cordata* | 0.762 | 0.762 | 53.957 | 31.851 | 11.141 | 2.192 |
| *Tilia platyphyllos* | 0.828 | 0.828 | 39.125 | 25.462 | 16.273 | 19.049 |
| *Sterculia apetala* | 0.940 | 0.937 | 5.968 | 32.188 | 3.139 | 55.414 |
| *Pachira aquatica* | 0.934 | 0.932 | 8.535 | 61.886 | 1.584 | 22.576 |
| *Cochlospermum religiosum* | 0.968 | 0.950 | 37.692 | 26.498 | 16.712 | 14.479 |
| *Helianthemum apenninum* | 0.922 | 0.922 | 39.319 | 5.738 | 2.275 | 50.991 |
| *Helianthemum ledifolium* | 0.955 | 0.953 | 50.725 | 8.633 | 2.101 | 33.390 |
| *Triplaris americana* | 0.944 | 0.941 | 3.565 | 30.886 | 7.376 | 56.156 |
| *Triplaris cumingiana* | 0.962 | 0.956 | 2.713 | 1.845 | 5.901 | 79.185 |
| *Atriplex nummularia* | 0.950 | 0.949 | 45.223 | 16.699 | 13.741 | 21.032 |
| *Phytolacca dioica* | 0.939 | 0.938 | 57.795 | 8.234 | 1.810 | 27.604 |
| *Philadelphus coronarius* | 0.799 | 0.799 | 56.187 | 26.217 | 7.993 | 9.105 |
| *Cornus sanguinea* | 0.771 | 0.771 | 57.278 | 22.755 | 6.674 | 13.151 |
| *Manilkara zapota* | 0.921 | 0.919 | 26.614 | 39.616 | 2.795 | 21.502 |
| *Daboecia cantabrica* | 0.984 | 0.983 | 37.502 | 25.077 | 5.906 | 30.721 |
| *Calluna vulgaris* | 0.696 | 0.696 | 40.760 | 34.047 | 6.835 | 17.512 |
| *Rhododendron ferrugineum* | 0.954 | 0.953 | 24.249 | 15.173 | 18.505 | 34.645 |
| *Rhododendron hirsutum* | 0.977 | 0.976 | 19.246 | 23.319 | 6.777 | 32.431 |
| *Rhododendron ponticum* | 0.910 | 0.909 | 33.608 | 24.574 | 14.056 | 25.149 |
| *Cinchona pubescens* | 0.968 | 0.966 | 3.100 | 2.708 | 1.550 | 62.471 |
| *Voacanga africana* | 0.962 | 0.955 | 17.713 | 28.335 | 6.131 | 46.392 |
| *Gomphocarpus fruticosus* | 0.911 | 0.910 | 54.013 | 5.013 | 1.072 | 39.504 |
| *Cordia alliodora* | 0.909 | 0.907 | 4.672 | 19.319 | 1.352 | 71.010 |
| *Solanum anguivi* | 0.913 | 0.910 | 10.538 | 12.058 | 3.131 | 62.101 |
| *Ligustrum vulgare* | 0.757 | 0.757 | 63.098 | 21.019 | 7.311 | 8.435 |
| *Fraxinus americana* | 0.894 | 0.894 | 15.683 | 44.423 | 13.830 | 25.899 |
| *Fraxinus angustifolia* | 0.870 | 0.870 | 59.792 | 12.043 | 2.084 | 25.758 |
| *Fraxinus excelsior* | 0.746 | 0.746 | 51.147 | 30.006 | 9.266 | 9.399 |
| *Fraxinus pennsylvanica* | 0.842 | 0.841 | 37.826 | 25.036 | 3.851 | 30.303 |
| *Fraxinus uhdei* | 0.958 | 0.953 | 28.931 | 5.086 | 7.868 | 30.808 |
| *Jacaranda mimosifolia* | 0.886 | 0.885 | 58.446 | 14.281 | 1.078 | 24.902 |
| *Catalpa speciosa* | 0.933 | 0.932 | 29.283 | 34.534 | 5.705 | 29.523 |
| *Tabebuia rosea* | 0.912 | 0.910 | 8.522 | 35.609 | 5.647 | 46.019 |
| *Spathodea campanulata* | 0.891 | 0.889 | 16.109 | 23.152 | 1.267 | 54.853 |
| *Lantana camara* | 0.738 | 0.738 | 60.431 | 31.562 | 0.147 | 5.504 |
| *Tectona grandis* | 0.902 | 0.900 | 41.378 | 44.106 | 5.081 | 8.402 |
| *Lonicera xylosteum* | 0.757 | 0.757 | 51.758 | 33.284 | 10.917 | 3.765 |
| *Symphoricarpos orbiculatus* | 0.919 | 0.919 | 22.989 | 42.536 | 6.698 | 26.331 |
| *Billardiera heterophylla* | 0.975 | 0.975 | 40.006 | 4.547 | 0.464 | 43.318 |
| *Quercus robur* | 0.726 | 0.726 | 57.762 | 30.283 | 9.019 | 2.511 |

**Table S2** Probability of invasion distribution of 250 alien woody species in Tianshan Potential National Park, Altai Kanas Potential National Park, Karamaili Potential National Park, and Xinjiang Nature Reserve under different climate scenarios. (The ranking method is based on the distribution probability of species in the current climate of Altai Kanas)

| species | current | | | | ssp245 | | | | ssp585 | | | |
| --- | --- | --- | --- | --- | --- | --- | --- | --- | --- | --- | --- | --- |
|  | Tianshan | Altai Kanas | Kalamari | NR of Xinjiang | Tianshan | Altai Kanas | Kalamari | NR of Xinjiang | Tianshan | Altai Kanas | Kalamari | NR of Xinjiang |
| *Acer negundo* | 0.289 | 0.386 | 0.212 | 0 | 0.426 | 0.484 | 0.214 | 0.068 | 0.528 | 0.528 | 0.168 | 0.08 |
| *Robinia pseudoacacia* | 0.145 | 0.287 | 0.212 | 0 | 0.183 | 0.298 | 0.169 | 0.039 | 0.205 | 0.264 | 0.088 | 0.036 |
| *Ribes nigrum* | 0.081 | 0.215 | 0.16 | 0 | 0.156 | 0.311 | 0.188 | 0.049 | 0.235 | 0.392 | 0.198 | 0.064 |
| *Amorpha fruticosa* | 0.062 | 0.194 | 0.133 | 0.026 | 0.142 | 0.284 | 0.172 | 0.034 | 0.243 | 0.359 | 0.171 | 0.043 |
| *Populus tremuloides* | 0.072 | 0.143 | 0.094 | 0.031 | 0.096 | 0.178 | 0.094 | 0.031 | 0.11 | 0.181 | 0.067 | 0.034 |
| *Betula pubescens* | 0.059 | 0.141 | 0.053 | 0 | 0.069 | 0.168 | 0.05 | 0.02 | 0.073 | 0.17 | 0.041 | 0.022 |
| *Populus nigra* | 0.05 | 0.14 | 0.145 | 0 | 0.093 | 0.216 | 0.19 | 0.045 | 0.142 | 0.269 | 0.197 | 0.05 |
| *Populus deltoides* | 0.026 | 0.133 | 0.107 | 0 | 0.081 | 0.261 | 0.196 | 0.026 | 0.162 | 0.356 | 0.223 | 0.035 |
| *Fraxinus pennsylvanica* | 0.012 | 0.117 | 0.083 | 0 | 0.032 | 0.197 | 0.117 | 0.014 | 0.06 | 0.262 | 0.129 | 0.019 |
| *Pinus contorta* | 0.131 | 0.1 | 0.022 | 0.027 | 0.148 | 0.12 | 0.015 | 0.027 | 0.157 | 0.123 | 0.008 | 0.03 |
| *Populus balsamifera* | 0.036 | 0.078 | 0.081 | 0 | 0.076 | 0.136 | 0.117 | 0.041 | 0.127 | 0.175 | 0.122 | 0.05 |
| *Quercus robur* | 0.017 | 0.077 | 0.066 | 0.01 | 0.032 | 0.108 | 0.071 | 0.013 | 0.049 | 0.137 | 0.073 | 0.015 |
| *Ulmus laevis* | 0.004 | 0.077 | 0.06 | 0 | 0.007 | 0.106 | 0.066 | 0.004 | 0.012 | 0.116 | 0.055 | 0.004 |
| *Acer platanoides* | 0.018 | 0.076 | 0.068 | 0 | 0.032 | 0.111 | 0.075 | 0.012 | 0.047 | 0.141 | 0.073 | 0.014 |
| *Genista tinctoria* | 0.007 | 0.074 | 0.046 | 0.004 | 0.015 | 0.117 | 0.055 | 0.005 | 0.025 | 0.15 | 0.052 | 0.007 |
| *Pinus banksiana* | 0.005 | 0.071 | 0.054 | 0.006 | 0.009 | 0.087 | 0.052 | 0.003 | 0.011 | 0.062 | 0.027 | 0.003 |
| *Alnus glutinosa* | 0.02 | 0.07 | 0.055 | 0 | 0.034 | 0.096 | 0.057 | 0.013 | 0.049 | 0.12 | 0.059 | 0.016 |
| *Berberis vulgaris* | 0.018 | 0.06 | 0.04 | 0.007 | 0.034 | 0.095 | 0.045 | 0.009 | 0.053 | 0.121 | 0.041 | 0.012 |
| *Lonicera xylosteum* | 0.011 | 0.06 | 0.033 | 0 | 0.02 | 0.091 | 0.035 | 0.006 | 0.029 | 0.115 | 0.033 | 0.007 |
| *Tilia cordata* | 0.01 | 0.06 | 0.042 | 0 | 0.019 | 0.089 | 0.046 | 0.006 | 0.028 | 0.112 | 0.043 | 0.007 |
| *Calluna vulgaris* | 0.043 | 0.058 | 0.013 | 0 | 0.047 | 0.065 | 0.011 | 0.016 | 0.05 | 0.064 | 0.011 | 0.019 |
| *Ribes aureum* | 0.012 | 0.057 | 0.05 | 0.016 | 0.039 | 0.156 | 0.118 | 0.021 | 0.086 | 0.215 | 0.135 | 0.025 |
| *Myrica gale* | 0.011 | 0.057 | 0.044 | 0 | 0.014 | 0.057 | 0.036 | 0.006 | 0.016 | 0.05 | 0.023 | 0.005 |
| *Ulmus americana* | 0.005 | 0.055 | 0.038 | 0 | 0.013 | 0.093 | 0.054 | 0.006 | 0.023 | 0.125 | 0.063 | 0.009 |
| *Picea abies* | 0.027 | 0.05 | 0.017 | 0.01 | 0.033 | 0.064 | 0.016 | 0.013 | 0.037 | 0.07 | 0.014 | 0.015 |
| *Ribes uva-crispa* | 0.013 | 0.046 | 0.035 | 0.006 | 0.022 | 0.067 | 0.037 | 0.007 | 0.032 | 0.085 | 0.034 | 0.009 |
| *Pinus ponderosa* | 0.07 | 0.044 | 0.025 | 0.023 | 0.126 | 0.07 | 0.025 | 0.025 | 0.198 | 0.103 | 0.022 | 0.029 |
| *Aesculus hippocastanum* | 0.012 | 0.044 | 0.037 | 0 | 0.023 | 0.07 | 0.047 | 0.01 | 0.038 | 0.092 | 0.048 | 0.011 |
| *Physocarpus opulifolius* | 0.004 | 0.039 | 0.028 | 0 | 0.008 | 0.059 | 0.033 | 0.003 | 0.014 | 0.07 | 0.03 | 0.004 |
| *Prunus domestica* | 0.014 | 0.038 | 0.034 | 0 | 0.026 | 0.056 | 0.038 | 0.012 | 0.041 | 0.072 | 0.039 | 0.013 |
| *Ligustrum vulgare* | 0.011 | 0.036 | 0.03 | 0 | 0.022 | 0.065 | 0.044 | 0.01 | 0.037 | 0.086 | 0.046 | 0.011 |
| *Spiraea alba* | 0.003 | 0.036 | 0.028 | 0 | 0.006 | 0.048 | 0.03 | 0.002 | 0.009 | 0.043 | 0.019 | 0.002 |
| *Fraxinus excelsior* | 0.01 | 0.035 | 0.025 | 0 | 0.016 | 0.051 | 0.027 | 0.006 | 0.023 | 0.066 | 0.027 | 0.008 |
| *Pseudotsuga menziesii* | 0.053 | 0.034 | 0.01 | 0.015 | 0.067 | 0.042 | 0.009 | 0.018 | 0.091 | 0.052 | 0.009 | 0.023 |
| *Ulmus minor* | 0.01 | 0.029 | 0.024 | 0 | 0.021 | 0.056 | 0.039 | 0.01 | 0.037 | 0.074 | 0.041 | 0.012 |
| *Cornus sanguinea* | 0.009 | 0.028 | 0.022 | 0 | 0.016 | 0.048 | 0.03 | 0.006 | 0.025 | 0.064 | 0.031 | 0.007 |
| *Philadelphus coronarius* | 0.006 | 0.028 | 0.02 | 0 | 0.013 | 0.048 | 0.027 | 0.005 | 0.023 | 0.063 | 0.029 | 0.006 |
| *Quercus rubra* | 0.004 | 0.028 | 0.021 | 0 | 0.009 | 0.046 | 0.028 | 0.004 | 0.016 | 0.057 | 0.028 | 0.005 |
| *Berberis thunbergii* | 0.004 | 0.022 | 0.018 | 0.003 | 0.009 | 0.04 | 0.025 | 0.003 | 0.016 | 0.048 | 0.023 | 0.004 |
| *Salix nigra* | 0.003 | 0.021 | 0.014 | 0 | 0.009 | 0.065 | 0.036 | 0.005 | 0.02 | 0.113 | 0.056 | 0.008 |
| *Pinus strobus* | 0.003 | 0.019 | 0.013 | 0.002 | 0.007 | 0.03 | 0.017 | 0.003 | 0.012 | 0.037 | 0.016 | 0.003 |
| *Juniperus virginiana* | 0.002 | 0.019 | 0.016 | 0.003 | 0.006 | 0.05 | 0.035 | 0.004 | 0.011 | 0.071 | 0.042 | 0.005 |
| *Juglans nigra* | 0.002 | 0.019 | 0.021 | 0 | 0.007 | 0.051 | 0.047 | 0.006 | 0.014 | 0.068 | 0.052 | 0.006 |
| *Fraxinus americana* | 0.003 | 0.018 | 0.023 | 0 | 0.009 | 0.034 | 0.036 | 0.007 | 0.017 | 0.047 | 0.04 | 0.008 |
| *Acer pseudoplatanus* | 0.012 | 0.016 | 0.008 | 0 | 0.016 | 0.022 | 0.009 | 0.007 | 0.022 | 0.026 | 0.009 | 0.008 |
| *Catalpa speciosa* | 0.002 | 0.016 | 0.024 | 0 | 0.01 | 0.061 | 0.071 | 0.013 | 0.023 | 0.088 | 0.086 | 0.015 |
| *Fagus sylvatica* | 0.01 | 0.012 | 0.006 | 0 | 0.013 | 0.017 | 0.007 | 0.005 | 0.018 | 0.021 | 0.007 | 0.007 |
| *Larix decidua* | 0.005 | 0.012 | 0.006 | 0.002 | 0.007 | 0.017 | 0.006 | 0.002 | 0.009 | 0.021 | 0.005 | 0.003 |
| *Ribes alpinum* | 0.005 | 0.01 | 0.004 | 0.002 | 0.007 | 0.014 | 0.003 | 0.003 | 0.01 | 0.017 | 0.003 | 0.004 |
| *Ribes multiflorum* | 0.009 | 0.009 | 0.008 | 0.008 | 0.021 | 0.02 | 0.013 | 0.009 | 0.038 | 0.034 | 0.016 | 0.011 |
| *Carpinus betulus* | 0.004 | 0.009 | 0.006 | 0 | 0.007 | 0.014 | 0.008 | 0.003 | 0.011 | 0.019 | 0.008 | 0.003 |
| *Sorbaria tomentosa* | 0.04 | 0.008 | 0.003 | 0 | 0.115 | 0.022 | 0.008 | 0.038 | 0.239 | 0.05 | 0.011 | 0.066 |
| *Cytisus scoparius* | 0.009 | 0.008 | 0.004 | 0.005 | 0.013 | 0.011 | 0.005 | 0.006 | 0.019 | 0.013 | 0.005 | 0.008 |
| *Juniperus occidentalis* | 0.036 | 0.007 | 0.004 | 0.008 | 0.045 | 0.008 | 0.004 | 0.007 | 0.064 | 0.01 | 0.002 | 0.008 |
| *Lantana camara* | 0.006 | 0.007 | 0.006 | 0 | 0.01 | 0.013 | 0.012 | 0.009 | 0.017 | 0.021 | 0.02 | 0.015 |
| *Tilia platyphyllos* | 0.003 | 0.007 | 0.004 | 0 | 0.005 | 0.011 | 0.005 | 0.002 | 0.007 | 0.014 | 0.005 | 0.003 |
| *Juglans cinerea* | 0 | 0.007 | 0.006 | 0 | 0.001 | 0.014 | 0.01 | 0.001 | 0.002 | 0.018 | 0.01 | 0.001 |
| *Pinus nigra* | 0.003 | 0.006 | 0.003 | 0.001 | 0.006 | 0.013 | 0.005 | 0.002 | 0.01 | 0.018 | 0.005 | 0.003 |
| *Salix repens* | 0.003 | 0.006 | 0.002 | 0 | 0.003 | 0.008 | 0.002 | 0.001 | 0.004 | 0.009 | 0.001 | 0.002 |
| *Spiraea douglasii* | 0.007 | 0.005 | 0.002 | 0 | 0.01 | 0.007 | 0.002 | 0.002 | 0.013 | 0.008 | 0.001 | 0.003 |
| *Amelanchier ovalis* | 0.002 | 0.005 | 0.001 | 0 | 0.003 | 0.008 | 0.002 | 0.001 | 0.004 | 0.011 | 0.002 | 0.001 |
| *Castanea sativa* | 0.005 | 0.004 | 0.002 | 0 | 0.006 | 0.006 | 0.002 | 0.003 | 0.01 | 0.008 | 0.003 | 0.003 |
| *Pyracantha coccinea* | 0.003 | 0.004 | 0.003 | 0 | 0.005 | 0.007 | 0.004 | 0.003 | 0.01 | 0.011 | 0.005 | 0.004 |
| *Quercus petraea* | 0.003 | 0.004 | 0.002 | 0 | 0.004 | 0.006 | 0.002 | 0.002 | 0.006 | 0.008 | 0.002 | 0.002 |
| *Fraxinus angustifolia* | 0.002 | 0.003 | 0.002 | 0 | 0.005 | 0.007 | 0.004 | 0.003 | 0.009 | 0.012 | 0.006 | 0.003 |
| *Styphnolobium japonicum* | 0.001 | 0.003 | 0.002 | 0.003 | 0.004 | 0.019 | 0.014 | 0.004 | 0.014 | 0.043 | 0.023 | 0.005 |
| *Symphoricarpos orbiculatus* | 0 | 0.003 | 0.004 | 0 | 0.001 | 0.016 | 0.016 | 0.001 | 0.002 | 0.03 | 0.025 | 0.002 |
| *Pinus monticola* | 0.011 | 0.002 | 0 | 0.003 | 0.013 | 0.003 | 0 | 0.003 | 0.018 | 0.003 | 0 | 0.003 |
| *Cedrus deodara* | 0.005 | 0.002 | 0.001 | 0.003 | 0.008 | 0.004 | 0.002 | 0.004 | 0.014 | 0.007 | 0.003 | 0.005 |
| *Pinus canariensis* | 0.004 | 0.002 | 0 | 0.001 | 0.006 | 0.003 | 0 | 0.001 | 0.01 | 0.005 | 0.001 | 0.002 |
| *Persea americana* | 0.004 | 0.002 | 0.001 | 0.002 | 0.006 | 0.003 | 0.002 | 0.003 | 0.009 | 0.004 | 0.002 | 0.004 |
| *Callistemon rigidus* | 0.004 | 0.002 | 0.001 | 0 | 0.005 | 0.003 | 0.001 | 0.003 | 0.008 | 0.004 | 0.001 | 0.004 |
| *Abies amabilis* | 0.004 | 0.002 | 0 | 0.001 | 0.003 | 0.001 | 0 | 0.001 | 0.003 | 0.001 | 0 | 0.001 |
| *Arenga pinnata* | 0.003 | 0.002 | 0.002 | 0.001 | 0.005 | 0.005 | 0.003 | 0.002 | 0.008 | 0.007 | 0.006 | 0.003 |
| *Azadirachta indica* | 0.003 | 0.002 | 0.001 | 0 | 0.004 | 0.003 | 0.002 | 0.004 | 0.007 | 0.005 | 0.005 | 0.007 |
| *Tamarindus indica* | 0.002 | 0.002 | 0.001 | 0.001 | 0.003 | 0.002 | 0.001 | 0.002 | 0.003 | 0.003 | 0.002 | 0.002 |
| *Abies alba* | 0.002 | 0.002 | 0.001 | 0.001 | 0.002 | 0.003 | 0 | 0.001 | 0.003 | 0.003 | 0 | 0.001 |
| *Cryptomeria japonica* | 0.001 | 0.002 | 0.002 | 0.002 | 0.002 | 0.006 | 0.006 | 0.002 | 0.006 | 0.009 | 0.007 | 0.002 |
| *Juglans ailanthifolia* | 0.001 | 0.002 | 0.001 | 0 | 0.001 | 0.004 | 0.001 | 0 | 0.002 | 0.006 | 0.001 | 0.001 |
| *Rosa pendulina* | 0.001 | 0.002 | 0 | 0 | 0.001 | 0.002 | 0 | 0 | 0.001 | 0.003 | 0 | 0 |
| *Ficus religiosa* | 0.003 | 0.001 | 0 | 0 | 0.005 | 0.001 | 0.001 | 0.003 | 0.007 | 0.001 | 0.001 | 0.004 |
| *Washingtonia filifera* | 0.002 | 0.001 | 0.001 | 0.001 | 0.004 | 0.002 | 0.004 | 0.002 | 0.011 | 0.005 | 0.012 | 0.005 |
| *Dalbergia sissoo* | 0.002 | 0.001 | 0.001 | 0.001 | 0.007 | 0.002 | 0.002 | 0.004 | 0.015 | 0.005 | 0.007 | 0.01 |
| *Washingtonia robusta* | 0.002 | 0.001 | 0.001 | 0.001 | 0.004 | 0.002 | 0.001 | 0.003 | 0.008 | 0.004 | 0.003 | 0.005 |
| *Spartium junceum* | 0.002 | 0.001 | 0.001 | 0.001 | 0.004 | 0.002 | 0.001 | 0.002 | 0.007 | 0.003 | 0.001 | 0.003 |
| *Fraxinus uhdei* | 0.002 | 0.001 | 0.001 | 0 | 0.003 | 0.002 | 0.001 | 0.001 | 0.005 | 0.002 | 0.002 | 0.002 |
| *Abies grandis* | 0.002 | 0.001 | 0 | 0.001 | 0.003 | 0.001 | 0 | 0.001 | 0.004 | 0.002 | 0 | 0.001 |
| *Helianthemum ledifolium* | 0.001 | 0.001 | 0.001 | 0 | 0.003 | 0.002 | 0.003 | 0.004 | 0.006 | 0.005 | 0.006 | 0.005 |
| *Delonix regia* | 0.001 | 0.001 | 0.001 | 0.002 | 0.002 | 0.002 | 0.003 | 0.004 | 0.004 | 0.003 | 0.005 | 0.008 |
| *Albizia lebbeck* | 0.001 | 0.001 | 0.001 | 0.001 | 0.001 | 0.002 | 0.002 | 0.002 | 0.003 | 0.003 | 0.005 | 0.005 |
| *Casuarina equisetifolia* | 0.001 | 0.001 | 0 | 0 | 0.003 | 0.001 | 0.001 | 0.002 | 0.006 | 0.003 | 0.002 | 0.004 |
| *Cassia fistula* | 0.001 | 0.001 | 0.001 | 0.001 | 0.002 | 0.001 | 0.001 | 0.002 | 0.003 | 0.002 | 0.003 | 0.004 |
| *Cupressus sempervirens* | 0.001 | 0.001 | 0 | 0.001 | 0.001 | 0.001 | 0.001 | 0.001 | 0.003 | 0.002 | 0.001 | 0.001 |
| *Ficus benghalensis* | 0.001 | 0.001 | 0 | 0 | 0.002 | 0.001 | 0.001 | 0.001 | 0.004 | 0.001 | 0.001 | 0.003 |
| *Chamaecyparis lawsoniana* | 0.001 | 0.001 | 0 | 0.001 | 0.001 | 0.001 | 0 | 0.001 | 0.002 | 0.001 | 0 | 0.001 |
| *Picea sitchensis* | 0.001 | 0.001 | 0 | 0 | 0.001 | 0.001 | 0 | 0 | 0.001 | 0.001 | 0 | 0.001 |
| *Ulex europaeus* | 0.001 | 0.001 | 0 | 0.001 | 0.002 | 0.001 | 0 | 0.001 | 0.002 | 0.001 | 0 | 0.001 |
| *Alnus rubra* | 0.001 | 0.001 | 0 | 0 | 0.001 | 0.001 | 0 | 0 | 0.001 | 0.001 | 0 | 0 |
| *Triplaris cumingiana* | 0.001 | 0.001 | 0 | 0 | 0.001 | 0.001 | 0 | 0 | 0.001 | 0.001 | 0 | 0 |
| *Pinus rigida* | 0 | 0.001 | 0.002 | 0.001 | 0.001 | 0.005 | 0.004 | 0.001 | 0.002 | 0.006 | 0.004 | 0 |
| *Pinus thunbergii* | 0 | 0.001 | 0 | 0.001 | 0.001 | 0.003 | 0.002 | 0.001 | 0.003 | 0.005 | 0.003 | 0.001 |
| *Genista germanica* | 0 | 0.001 | 0.001 | 0 | 0.001 | 0.003 | 0.001 | 0 | 0.001 | 0.004 | 0.001 | 0 |
| *Castanea crenata* | 0 | 0.001 | 0.001 | 0 | 0.001 | 0.002 | 0.002 | 0.001 | 0.001 | 0.003 | 0.002 | 0.001 |
| *Larix kaempferi* | 0 | 0.001 | 0.001 | 0 | 0.001 | 0.003 | 0.001 | 0 | 0.002 | 0.003 | 0.001 | 0 |
| *Pimenta dioica* | 0 | 0.001 | 0.001 | 0 | 0 | 0.002 | 0.001 | 0 | 0.001 | 0.002 | 0.002 | 0 |
| *Pinus parviflora* | 0 | 0.001 | 0.001 | 0 | 0.001 | 0.002 | 0.001 | 0 | 0.002 | 0.002 | 0.001 | 0 |
| *Eucalyptus benthamii* | 0 | 0.001 | 0.001 | 0 | 0 | 0.001 | 0.001 | 0 | 0.001 | 0.002 | 0.001 | 0 |
| *Quercus cerris* | 0 | 0.001 | 0 | 0 | 0.001 | 0.001 | 0.001 | 0.001 | 0.002 | 0.002 | 0.001 | 0.001 |
| *Eucalyptus megacornuta* | 0 | 0.001 | 0 | 0 | 0 | 0.001 | 0.001 | 0 | 0 | 0.002 | 0.001 | 0 |
| *Rhododendron ponticum* | 0 | 0.001 | 0 | 0 | 0.001 | 0.001 | 0.001 | 0 | 0.001 | 0.002 | 0.001 | 0 |
| *Cytisus nigricans* | 0 | 0.001 | 0 | 0 | 0 | 0.002 | 0.001 | 0 | 0.001 | 0.002 | 0 | 0 |
| *Erythrina poeppigiana* | 0 | 0.001 | 0.001 | 0 | 0 | 0.001 | 0.001 | 0 | 0.001 | 0.001 | 0.001 | 0 |
| *Eucalyptus robusta* | 0.002 | 0 | 0 | 0 | 0.004 | 0.001 | 0 | 0.001 | 0.007 | 0.001 | 0 | 0.002 |
| *Schinus molle* | 0.001 | 0 | 0.001 | 0 | 0.002 | 0.001 | 0.001 | 0.005 | 0.004 | 0.002 | 0.002 | 0.008 |
| *Quercus coccifera* | 0.001 | 0 | 0 | 0 | 0.001 | 0.001 | 0 | 0.001 | 0.003 | 0.002 | 0.001 | 0.001 |
| *Eucalyptus camaldulensis* | 0.001 | 0 | 0 | 0 | 0.002 | 0.001 | 0.001 | 0 | 0.003 | 0.002 | 0.001 | 0.001 |
| *Pinus halepensis* | 0.001 | 0 | 0 | 0 | 0.001 | 0.001 | 0 | 0.001 | 0.002 | 0.001 | 0.001 | 0.001 |
| *Hippocrepis emerus* | 0.001 | 0 | 0 | 0 | 0.001 | 0.001 | 0 | 0 | 0.001 | 0.001 | 0 | 0 |
| *Galphimia glauca* | 0.001 | 0 | 0 | 0 | 0.001 | 0 | 0 | 0 | 0.002 | 0.001 | 0 | 0 |
| *Eucalyptus globulus* | 0.001 | 0 | 0 | 0 | 0.001 | 0 | 0 | 0.001 | 0.002 | 0.001 | 0 | 0.001 |
| *Helianthemum apenninum* | 0.001 | 0 | 0 | 0 | 0.001 | 0.001 | 0 | 0 | 0.001 | 0.001 | 0 | 0 |
| *Cinchona pubescens* | 0.001 | 0 | 0 | 0 | 0.001 | 0 | 0 | 0 | 0.001 | 0.001 | 0 | 0 |
| *Pinus oocarpa* | 0.001 | 0 | 0 | 0 | 0.001 | 0 | 0 | 0 | 0.001 | 0 | 0 | 0 |
| *Berberis microphylla* | 0.001 | 0 | 0 | 0.001 | 0.001 | 0 | 0 | 0 | 0.001 | 0 | 0 | 0 |
| *Acaciella glauca* | 0.001 | 0 | 0 | 0 | 0.001 | 0 | 0 | 0 | 0.001 | 0 | 0 | 0 |
| *Tephrosia candida* | 0.001 | 0 | 0 | 0 | 0.001 | 0 | 0 | 0.002 | 0 | 0 | 0 | 0.002 |
| *Pinus taeda* | 0 | 0 | 0 | 0 | 0 | 0.001 | 0.001 | 0 | 0 | 0.002 | 0.002 | 0 |
| *Pinus luchuensis* | 0 | 0 | 0 | 0 | 0 | 0 | 0.001 | 0 | 0 | 0.001 | 0.001 | 0 |
| *Annona squamosa* | 0 | 0 | 0 | 0 | 0 | 0 | 0 | 0 | 0.001 | 0.001 | 0.001 | 0.001 |
| *Cinnamomum verum* | 0 | 0 | 0 | 0 | 0 | 0.001 | 0.001 | 0 | 0.001 | 0.001 | 0.001 | 0.001 |
| *Grevillea robusta* | 0 | 0 | 0 | 0 | 0 | 0 | 0 | 0 | 0 | 0.001 | 0.001 | 0.001 |
| *Mimosa pigra* | 0 | 0 | 0 | 0 | 0 | 0 | 0 | 0 | 0.001 | 0.001 | 0.001 | 0.001 |
| *Acacia iteaphylla* | 0 | 0 | 0 | 0 | 0 | 0 | 0 | 0 | 0 | 0.001 | 0.001 | 0 |
| *Averrhoa carambola* | 0 | 0 | 0 | 0 | 0 | 0 | 0 | 0 | 0 | 0.001 | 0.001 | 0 |
| *Metrosideros excelsa* | 0 | 0 | 0 | 0 | 0 | 0 | 0 | 0 | 0 | 0.001 | 0.001 | 0 |
| *Jacaranda mimosifolia* | 0 | 0 | 0 | 0 | 0.001 | 0.001 | 0 | 0 | 0.001 | 0.001 | 0.001 | 0.001 |
| *Pinus caribaea* | 0 | 0 | 0 | 0 | 0 | 0 | 0 | 0 | 0 | 0.001 | 0 | 0 |
| *Pinus pinaster* | 0 | 0 | 0 | 0 | 0 | 0 | 0 | 0 | 0.001 | 0.001 | 0 | 0 |
| *Ptychosperma macarthurii* | 0 | 0 | 0 | 0 | 0.001 | 0 | 0 | 0 | 0.001 | 0.001 | 0 | 0.001 |
| *Genista pilosa* | 0 | 0 | 0 | 0 | 0 | 0 | 0 | 0 | 0 | 0.001 | 0 | 0 |
| *Genista sagittalis* | 0 | 0 | 0 | 0 | 0 | 0 | 0 | 0 | 0.001 | 0.001 | 0 | 0 |
| *Rhamnus alaternus* | 0 | 0 | 0 | 0 | 0.001 | 0.001 | 0 | 0 | 0.001 | 0.001 | 0 | 0 |
| *Hevea brasiliensis* | 0 | 0 | 0 | 0 | 0 | 0 | 0 | 0 | 0.001 | 0.001 | 0 | 0 |
| *Aleurites moluccanus* | 0 | 0 | 0 | 0 | 0 | 0 | 0 | 0 | 0.001 | 0.001 | 0 | 0 |
| *Staphylea pinnata* | 0 | 0 | 0 | 0 | 0 | 0 | 0 | 0 | 0 | 0.001 | 0 | 0 |
| *Solanum anguivi* | 0 | 0 | 0 | 0 | 0.001 | 0 | 0 | 0 | 0.001 | 0.001 | 0 | 0.001 |
| *Phytelephas macrocarpa* | 0 | 0 | 0 | 0 | 0 | 0 | 0 | 0 | 0 | 0 | 0.001 | 0 |
| *Atriplex nummularia* | 0 | 0 | 0 | 0 | 0 | 0 | 0 | 0 | 0 | 0 | 0.001 | 0 |
| *Abies nordmanniana* | 0 | 0 | 0 | 0 | 0 | 0 | 0 | 0 | 0 | 0 | 0 | 0 |
| *Abies procera* | 0 | 0 | 0 | 0 | 0 | 0 | 0 | 0 | 0 | 0 | 0 | 0 |
| *Abies sachalinensis* | 0 | 0 | 0 | 0 | 0 | 0 | 0 | 0 | 0 | 0 | 0 | 0 |
| *Pinus elliottii* | 0 | 0 | 0 | 0 | 0 | 0 | 0 | 0 | 0 | 0 | 0 | 0 |
| *Pinus glabra* | 0 | 0 | 0 | 0 | 0 | 0 | 0 | 0 | 0 | 0 | 0 | 0 |
| *Pinus patula* | 0 | 0 | 0 | 0 | 0 | 0 | 0 | 0 | 0 | 0 | 0 | 0 |
| *Pinus pinea* | 0 | 0 | 0 | 0 | 0 | 0 | 0 | 0 | 0 | 0 | 0 | 0 |
| *Pinus radiata* | 0 | 0 | 0 | 0 | 0 | 0 | 0 | 0 | 0 | 0 | 0 | 0 |
| *Borassus flabellifer* | 0 | 0 | 0 | 0 | 0 | 0 | 0 | 0 | 0 | 0 | 0 | 0 |
| *Roystonea oleracea* | 0 | 0 | 0 | 0 | 0 | 0 | 0 | 0 | 0 | 0 | 0 | 0 |
| *Elaeis guineensis* | 0 | 0 | 0 | 0 | 0 | 0 | 0 | 0 | 0 | 0 | 0 | 0 |
| *Archontophoenix cunninghamiana* | 0 | 0 | 0 | 0 | 0 | 0 | 0 | 0 | 0 | 0 | 0 | 0 |
| *Ptychosperma elegans* | 0 | 0 | 0 | 0 | 0 | 0 | 0 | 0 | 0 | 0 | 0 | 0 |
| *Berberis darwinii* | 0 | 0 | 0 | 0 | 0 | 0 | 0 | 0 | 0 | 0 | 0 | 0 |
| *Hakea salicifolia* | 0 | 0 | 0 | 0 | 0 | 0 | 0 | 0 | 0 | 0 | 0 | 0 |
| *Grevillea banksii* | 0 | 0 | 0 | 0 | 0 | 0 | 0 | 0 | 0 | 0 | 0 | 0 |
| *Dillenia suffruticosa* | 0 | 0 | 0 | 0 | 0 | 0 | 0 | 0 | 0 | 0 | 0 | 0 |
| *Copaifera officinalis* | 0 | 0 | 0 | 0 | 0 | 0 | 0 | 0 | 0 | 0 | 0 | 0 |
| *Senna multijuga* | 0 | 0 | 0 | 0 | 0 | 0 | 0 | 0 | 0 | 0 | 0 | 0 |
| *Senna siamea* | 0 | 0 | 0 | 0 | 0 | 0 | 0 | 0 | 0 | 0 | 0 | 0 |
| *Senna spectabilis* | 0 | 0 | 0 | 0 | 0 | 0 | 0 | 0 | 0 | 0 | 0 | 0 |
| *Schizolobium parahyba* | 0 | 0 | 0 | 0 | 0 | 0 | 0 | 0 | 0 | 0 | 0 | 0 |
| *Acacia adunca* | 0 | 0 | 0 | 0 | 0 | 0 | 0 | 0 | 0 | 0 | 0 | 0 |
| *Acacia decurrens* | 0 | 0 | 0 | 0 | 0 | 0 | 0 | 0 | 0 | 0 | 0 | 0 |
| *Acacia elata* | 0 | 0 | 0 | 0 | 0 | 0 | 0 | 0 | 0 | 0 | 0 | 0 |
| *Acacia fimbriata* | 0 | 0 | 0 | 0 | 0 | 0 | 0 | 0 | 0 | 0 | 0 | 0 |
| *Acacia holosericea* | 0 | 0 | 0 | 0 | 0 | 0 | 0 | 0 | 0 | 0 | 0 | 0 |
| *Acacia implexa* | 0 | 0 | 0 | 0 | 0 | 0 | 0 | 0 | 0 | 0 | 0 | 0 |
| *Acacia mangium* | 0 | 0 | 0 | 0 | 0 | 0 | 0 | 0 | 0 | 0 | 0 | 0 |
| *Acacia melanoxylon* | 0 | 0 | 0 | 0 | 0 | 0 | 0 | 0 | 0 | 0 | 0 | 0 |
| *Acacia paradoxa* | 0 | 0 | 0 | 0 | 0 | 0 | 0 | 0 | 0 | 0 | 0 | 0 |
| *Acacia podalyriifolia* | 0 | 0 | 0 | 0 | 0 | 0 | 0 | 0 | 0 | 0 | 0 | 0 |
| *Myroxylon balsamum* | 0 | 0 | 0 | 0 | 0 | 0 | 0 | 0 | 0 | 0 | 0 | 0 |
| *Chamaecytisus ratisbonensis* | 0 | 0 | 0 | 0 | 0 | 0 | 0 | 0 | 0 | 0 | 0 | 0 |
| *Tipuana tipu* | 0 | 0 | 0 | 0 | 0 | 0 | 0 | 0 | 0 | 0 | 0 | 0 |
| *Gliricidia sepium* | 0 | 0 | 0 | 0 | 0 | 0 | 0 | 0 | 0.001 | 0 | 0 | 0 |
| *Medicago arborea* | 0 | 0 | 0 | 0 | 0 | 0 | 0 | 0 | 0 | 0 | 0 | 0 |
| *Polygala myrtifolia* | 0 | 0 | 0 | 0 | 0 | 0 | 0 | 0 | 0 | 0 | 0 | 0 |
| *Rubus moluccanus* | 0 | 0 | 0 | 0 | 0 | 0 | 0 | 0 | 0 | 0 | 0 | 0 |
| *Amelanchier lamarckii* | 0 | 0 | 0 | 0 | 0 | 0 | 0 | 0 | 0 | 0 | 0 | 0 |
| *Cotoneaster simonsii* | 0 | 0 | 0 | 0 | 0 | 0 | 0 | 0 | 0 | 0 | 0 | 0 |
| *Artocarpus altilis* | 0 | 0 | 0 | 0 | 0 | 0 | 0 | 0 | 0 | 0 | 0 | 0 |
| *Artocarpus heterophyllus* | 0 | 0 | 0 | 0 | 0 | 0 | 0 | 0 | 0.001 | 0 | 0 | 0 |
| *Ficus rubiginosa* | 0 | 0 | 0 | 0 | 0 | 0 | 0 | 0 | 0 | 0 | 0 | 0 |
| *Cecropia peltata* | 0 | 0 | 0 | 0 | 0 | 0 | 0 | 0 | 0 | 0 | 0 | 0 |
| *Casuarina cunninghamiana* | 0 | 0 | 0 | 0 | 0 | 0 | 0 | 0 | 0 | 0 | 0 | 0 |
| *Casuarina glauca* | 0 | 0 | 0 | 0 | 0 | 0 | 0 | 0 | 0 | 0 | 0 | 0 |
| *Allocasuarina littoralis* | 0 | 0 | 0 | 0 | 0 | 0 | 0 | 0 | 0 | 0 | 0 | 0 |
| *Pentadesma butyracea* | 0 | 0 | 0 | 0 | 0 | 0 | 0 | 0 | 0 | 0 | 0 | 0 |
| *Mammea americana* | 0 | 0 | 0 | 0 | 0 | 0 | 0 | 0 | 0 | 0 | 0 | 0 |
| *Dovyalis caffra* | 0 | 0 | 0 | 0 | 0 | 0 | 0 | 0 | 0 | 0 | 0 | 0 |
| *Reutealis trisperma* | 0 | 0 | 0 | 0 | 0 | 0 | 0 | 0 | 0 | 0 | 0 | 0 |
| *Garcia nutans* | 0 | 0 | 0 | 0 | 0 | 0 | 0 | 0 | 0.001 | 0 | 0 | 0 |
| *Hura crepitans* | 0 | 0 | 0 | 0 | 0 | 0 | 0 | 0 | 0 | 0 | 0 | 0 |
| *Melianthus major* | 0 | 0 | 0 | 0 | 0 | 0 | 0 | 0 | 0 | 0 | 0 | 0 |
| *Sonneratia apetala* | 0 | 0 | 0 | 0 | 0 | 0 | 0 | 0 | 0 | 0 | 0 | 0 |
| *Callistemon linearis* | 0 | 0 | 0 | 0 | 0 | 0 | 0 | 0 | 0 | 0 | 0 | 0 |
| *Callistemon viminalis* | 0 | 0 | 0 | 0 | 0 | 0 | 0 | 0 | 0 | 0 | 0 | 0 |
| *Melaleuca hypericifolia* | 0 | 0 | 0 | 0 | 0 | 0 | 0 | 0 | 0 | 0 | 0 | 0 |
| *Corymbia citriodora* | 0 | 0 | 0 | 0 | 0 | 0 | 0 | 0 | 0 | 0 | 0 | 0 |
| *Corymbia maculata* | 0 | 0 | 0 | 0 | 0 | 0 | 0 | 0 | 0 | 0 | 0 | 0 |
| *Corymbia torelliana* | 0 | 0 | 0 | 0 | 0 | 0 | 0 | 0 | 0 | 0 | 0 | 0 |
| *Eucalyptus botryoides* | 0 | 0 | 0 | 0 | 0 | 0 | 0 | 0 | 0 | 0 | 0 | 0 |
| *Eucalyptus brassiana* | 0 | 0 | 0 | 0 | 0 | 0 | 0 | 0 | 0 | 0 | 0 | 0 |
| *Eucalyptus cinerea* | 0 | 0 | 0 | 0 | 0 | 0 | 0 | 0 | 0 | 0 | 0 | 0 |
| *Eucalyptus cladocalyx* | 0 | 0 | 0 | 0 | 0 | 0 | 0 | 0 | 0 | 0 | 0 | 0 |
| *Eucalyptus cloeziana* | 0 | 0 | 0 | 0 | 0 | 0 | 0 | 0 | 0 | 0 | 0 | 0 |
| *Eucalyptus diversicolor* | 0 | 0 | 0 | 0 | 0 | 0 | 0 | 0 | 0 | 0 | 0 | 0 |
| *Eucalyptus gomphocephala* | 0 | 0 | 0 | 0 | 0 | 0 | 0 | 0 | 0 | 0 | 0 | 0 |
| *Eucalyptus grandis* | 0 | 0 | 0 | 0 | 0 | 0 | 0 | 0 | 0 | 0 | 0 | 0 |
| *Eucalyptus gunnii* | 0 | 0 | 0 | 0 | 0 | 0 | 0 | 0 | 0 | 0 | 0 | 0 |
| *Eucalyptus lehmannii* | 0 | 0 | 0 | 0 | 0 | 0 | 0 | 0 | 0 | 0 | 0 | 0 |
| *Eucalyptus nitens* | 0 | 0 | 0 | 0 | 0 | 0 | 0 | 0 | 0 | 0 | 0 | 0 |
| *Eucalyptus pellita* | 0 | 0 | 0 | 0 | 0 | 0 | 0 | 0 | 0 | 0 | 0 | 0 |
| *Eucalyptus saligna* | 0 | 0 | 0 | 0 | 0 | 0 | 0 | 0 | 0 | 0 | 0 | 0 |
| *Eucalyptus sideroxylon* | 0 | 0 | 0 | 0 | 0 | 0 | 0 | 0 | 0 | 0 | 0 | 0 |
| *Eucalyptus tereticornis* | 0 | 0 | 0 | 0 | 0 | 0 | 0 | 0 | 0 | 0 | 0 | 0 |
| *Eucalyptus urophylla* | 0 | 0 | 0 | 0 | 0 | 0 | 0 | 0 | 0 | 0 | 0 | 0 |
| *Eucalyptus viminalis* | 0 | 0 | 0 | 0 | 0 | 0 | 0 | 0 | 0 | 0 | 0 | 0 |
| *Syzygium malaccense* | 0 | 0 | 0 | 0 | 0 | 0 | 0 | 0 | 0 | 0 | 0 | 0 |
| *Syzygium paniculatum* | 0 | 0 | 0 | 0 | 0 | 0 | 0 | 0 | 0 | 0 | 0 | 0 |
| *Psidium guineense* | 0 | 0 | 0 | 0 | 0 | 0 | 0 | 0 | 0 | 0 | 0 | 0 |
| *Heterotis rotundifolia* | 0 | 0 | 0 | 0 | 0 | 0 | 0 | 0 | 0 | 0 | 0 | 0 |
| *Melicoccus bijugatus* | 0 | 0 | 0 | 0 | 0 | 0 | 0 | 0 | 0 | 0 | 0 | 0 |
| *Cedrela odorata* | 0 | 0 | 0 | 0 | 0 | 0 | 0 | 0 | 0 | 0 | 0 | 0 |
| *Triumfetta bogotensis* | 0 | 0 | 0 | 0 | 0 | 0 | 0 | 0 | 0 | 0 | 0 | 0 |
| *Sterculia apetala* | 0 | 0 | 0 | 0 | 0 | 0 | 0 | 0 | 0 | 0 | 0 | 0 |
| *Pachira aquatica* | 0 | 0 | 0 | 0 | 0 | 0 | 0 | 0 | 0 | 0 | 0 | 0 |
| *Cochlospermum religiosum* | 0 | 0 | 0 | 0 | 0 | 0 | 0 | 0 | 0 | 0 | 0 | 0 |
| *Triplaris americana* | 0 | 0 | 0 | 0 | 0 | 0 | 0 | 0 | 0 | 0 | 0 | 0 |
| *Phytolacca dioica* | 0 | 0 | 0 | 0 | 0 | 0 | 0 | 0 | 0 | 0 | 0 | 0 |
| *Manilkara zapota* | 0 | 0 | 0 | 0 | 0 | 0 | 0 | 0 | 0 | 0 | 0 | 0 |
| *Daboecia cantabrica* | 0 | 0 | 0 | 0 | 0 | 0 | 0 | 0 | 0 | 0 | 0 | 0 |
| *Rhododendron ferrugineum* | 0 | 0 | 0 | 0 | 0 | 0 | 0 | 0 | 0 | 0 | 0 | 0 |
| *Rhododendron hirsutum* | 0 | 0 | 0 | 0 | 0 | 0 | 0 | 0 | 0 | 0 | 0 | 0 |
| *Voacanga africana* | 0 | 0 | 0 | 0 | 0 | 0 | 0 | 0 | 0 | 0 | 0 | 0 |
| *Gomphocarpus fruticosus* | 0 | 0 | 0 | 0 | 0 | 0 | 0 | 0 | 0 | 0 | 0 | 0 |
| *Cordia alliodora* | 0 | 0 | 0 | 0 | 0 | 0 | 0 | 0 | 0 | 0 | 0 | 0 |
| *Tabebuia rosea* | 0 | 0 | 0 | 0 | 0 | 0 | 0 | 0 | 0 | 0 | 0 | 0 |
| *Spathodea campanulata* | 0 | 0 | 0 | 0 | 0 | 0 | 0 | 0 | 0 | 0 | 0 | 0 |
| *Tectona grandis* | 0 | 0 | 0 | 0 | 0 | 0 | 0 | 0 | 0 | 0 | 0 | 0 |
| *Billardiera heterophylla* | 0 | 0 | 0 | 0 | 0 | 0 | 0 | 0 | 0 | 0 | 0 | 0 |
